# Generation of a more efficient prime editor 2 by addition of the Rad51 DNA-binding domain

**DOI:** 10.1038/s41467-021-25928-2

**Published:** 2021-09-23

**Authors:** Myungjae Song, Jung Min Lim, Seonwoo Min, Jeong-Seok Oh, Dong Young Kim, Jae-Sung Woo, Hiroshi Nishimasu, Sung-Rae Cho, Sungroh Yoon, Hyongbum Henry Kim

**Affiliations:** 1 Department of Pharmacology, Yonsei University College of Medicine, Seoul, Republic of Korea; 2 Graduate School of Medical Science, Brain Korea 21 Plus Project for Medical Sciences, Yonsei University College of Medicine, Seoul, Republic of Korea; 3 Department of Electrical and Computer Engineering, Seoul National University, Seoul, Republic of Korea; 4 Department of Life Sciences, Korea University, Seoul, Republic of Korea; 5 Structural Biology Division, Research Center for Advanced Science and Technology, The University of Tokyo, Tokyo, Japan; 6 Department and Research Institute of Rehabilitation Medicine, Yonsei University College of Medicine, Seoul, Republic of Korea; 7 Graduate Program of Nano Science and Technology, Yonsei University, Seoul, Republic of Korea; 8 Rehabilitation Institute of Neuromuscular Disease, Yonsei University College of Medicine, Seoul, Republic of Korea; 9 Interdisciplinary Program in Artificial Intelligence, Seoul National University, Seoul, Republic of Korea; 10 Center for Nanomedicine, Institute for Basic Science (IBS), Seoul, Republic of Korea; 11 Graduate Program of Nano Biomedical Engineering (NanoBME), Advanced Science Institute, Yonsei University, Seoul, Republic of Korea; 12 Severance Biomedical Science Institute, Yonsei University College of Medicine, Seoul, Republic of Korea

**Keywords:** Genetic engineering, CRISPR-Cas9 genome editing, Molecular engineering

## Abstract

Although prime editing is a promising genome editing method, the efficiency of prime editor 2 (PE2) is often insufficient. Here we generate a more efficient variant of PE2, named hyPE2, by adding the Rad51 DNA-binding domain. When tested at endogenous sites, hyPE2 shows a median of 1.5- or 1.4- fold (range, 0.99- to 2.6-fold) higher efficiencies than PE2; furthermore, at sites where PE2-induced prime editing is very inefficient (efficiency < 1%), hyPE2 enables prime editing with efficiencies ranging from 1.1% to 2.9% at up to 34% of target sequences, potentially facilitating prime editing applications.

## Abstract (web summary)

While prime editing is a promising technology, PE2 systems often have low efficiency. Here the authors fuse a Rad51 DNA-binding domain to create hyPE2 with improved editing efficiency.

## Introduction

Prime editing can induce any small-sized genetic change, including insertions, deletions, all 12 possible point mutations, and combinations of these changes, without requiring donor DNA or double-strand breaks^1,2^. Prime editor (PE) 2 is composed of a Cas9-nickase reverse-transcriptase (RT) fusion protein and a prime-editing guide RNA (pegRNA)^1^. PE2 has been shown to induce prime editing in various species and cell types, including human cells^1–8^. However, depending on the target sequence and the used cell type, the efficiency of PE2 is often insufficient^1,2^. To further improve prime-editing efficiency, a single-guide RNA (sgRNA) can be added to PE2, resulting in PE3 and PE3b. PE3 and PE3b often, but not always, exhibit higher prime-editing efficiency at the expense of a higher risk of generating unintended indels^1,9–12^.

Enhancing the efficiency of prime editing would clearly facilitate its applications. When the Cas9-nickase domain and the pegRNA bind to the target sequence, a single-stranded DNA (ssDNA) is generated and used as a primer for reverse transcription of the pegRNA RT-template region. We postulated that the stabilization of the ssDNA by the addition of a ssDNA-binding protein domain (ssDBD) might enhance the efficiency of prime editing, although we could not rule out the possibility that the ssDBD might prevent binding of the pegRNA to the ssDNA, blocking the reverse transcription of the pegRNA RT-template region. However, the addition of a ssDBD has not been tested as a method to enhance prime-editing efficiency.

In this work, we generate hyPE2, a variant of PE2 that contains the Rad51 DBD between the Cas9 H840A nickase and RT domains, connected by linkers that are 16 and 33 amino acids in length. HyPE2 shows enhanced prime-editing efficiencies by a median of 1.5- or 1.4- fold (range, 0.99- to 2.6-fold) at endogenous sites where PE2-induced prime-editing efficiencies are higher than 1%. At target sequences where PE2-induced prime editing efficiencies are lower than 1%, hyPE2 enabled prime-editing with efficiencies ranging from 1.1% to 2.9% at up to 34% of target sequences. We test two ssDBDs, three positions for the ssDBD addition, and seven combinations of linkers to identify the most efficient PE2 variant. Furthermore, we also developed a computational model, named PEselector, that predicts the fold increase of hyPE2-induced prime-editing efficiencies as compared to those of PE2 and provide it as a web tool at http://deepcrispr.info/PEselector. We expect that hyPE2 and PEselector will promote successful applications of prime editing.

## Results

### Development of hyPE2

**Fig. 1:**
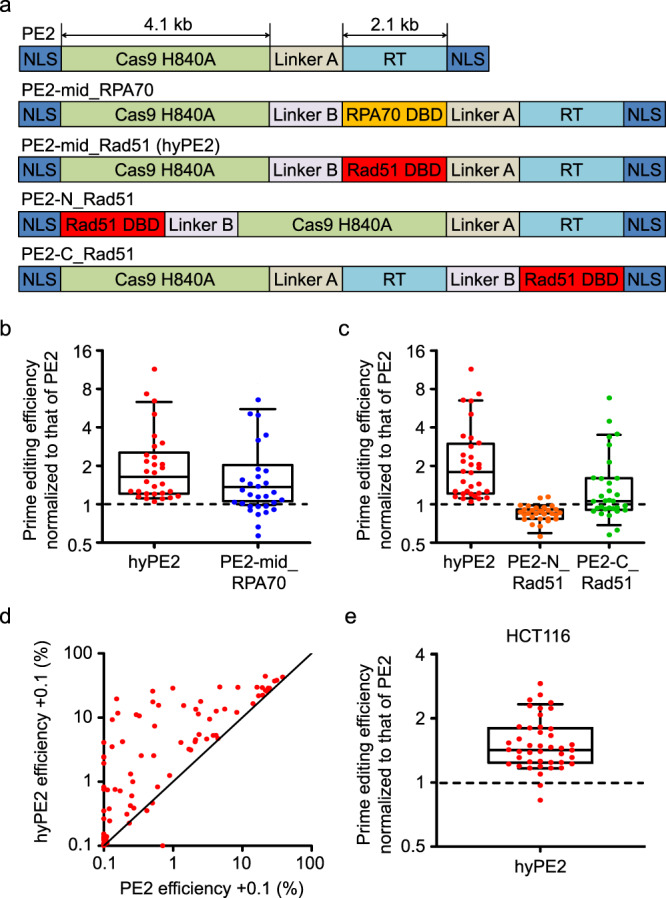
Design and activity evaluation of PE2 variants containing single-strand DNA-binding domains. **a** The structure of the PE2 variants containing the single-strand DNA-binding domains (ssDBDs) RPA70 DBD and Rad51 DBD. NLS nuclear-localization signal, RT reverse transcriptase. **b**, **c** Prime editing efficiencies of PE2 variants normalized to the efficiency of PE2 at the same target sequences, which had been lentivirally integrated in HEK293T cells. The number of target sequences *n* = 30 (**b**) and 32 (**c**). **d** Comparison of the prime-editing efficiencies of PE2 and hyPE2 in HEK293T cells. The black line indicates *y* = *x*. The data points represent the average prime-editing efficiency from three biological replicates at each target sequence. In all, 0.1% was added to all efficiency values so that log scales could be used for both the *x-* and *y* axes. The number of target sequences *n* = 88. **e** Prime editing efficiencies of PE2 variants normalized to the efficiency of PE2 at the same target sequences, which had been lentivirally integrated in HCT116 cells. The number of target sequences *n* = 43. **b**, **c**, **e** PegRNAs that resulted in PE2-directed prime-editing efficiencies higher than 1% are shown. Data of minimum-to-maximum values are presented. For the boxes, the top, middle, and bottom lines represent the 25th, 50th, and 75th percentiles, respectively. The whiskers indicate the 10th- and 90th-percentile values. Source data are provided as a Source Data file.

To test whether the addition of a DBD in PE2 would enhance the prime-editing efficiency, we first added either Rad51 DBD or RPA70-C, both of which are ssDBDs that have previously been shown to increase the efficiency of cytosine-base editors^13^, between the Cas9 nickase and RT domains of PE2, generating PE2 variants named PE2-mid_Rad51 (hyperPE2 or for brevity, hyPE2) and PE2-mid_RPA70, respectively (Fig. 1a). Testing these variants at one or two target sequences will not allow generalized conclusions to be made about the activities of these variants because prime-editing efficiencies vary greatly, depending on the target sequence^1,2^. Previously, we determined the activities of a total of 54,836 pairs of pegRNA-encoding and target sequences (hereafter, for brevity, pairs) using a lentiviral library of these pairs^2^. We randomly selected 107 plasmids from the previously used plasmid library 1 of 48,000 pairs^2^. From this plasmid library of 107 pairs (Supplementary Table 1), we generated a lentiviral library, which was transduced into HEK293T cells to make a cell library, which was named library A. In library A, the target sequences and the corresponding pegRNA-encoding sequences were lentivirally integrated into the genome. We delivered plasmids encoding hyPE2, PE2-mid_RPA70, or PE2 into the cell library, after which the prime-editing efficiencies were determined using deep sequencing. We observed high correlations between biological replicates (Supplementary Fig. 1) as we did in the previous study^2^ and the averages of prime-editing efficiencies from three biological replicates were obtained and used for analyses. From the initial 107 pairs, we excluded 24 pairs because of insufficient deep-sequencing read counts (<100 reads) and 19 pairs because of 0% editing efficiency with PE2, which prevents the normalization of hyPE2 efficiency to that of PE2. The median-fold increases in the prime editing efficiencies normalized to that of PE for the remaining 64 pairs were 2.4-fold (range, 0- to 360-fold) for hyPE2 and 1.5-fold for PE2-mid_RPA70 (range, 0- to 232-fold) (Supplementary Fig. 2). It appeared that the median fold increases were higher for pairs showing <1% editing efficiency with PE2, compared with pairs showing >1% efficiency with PE2. Given that calculations of the fold increases of these variants are more error-prone when the PE2 efficiencies at the target sequences are too low, we removed an additional 34 target sequences that showed PE2 efficiencies lower than 1% from the subsequent calculations of the fold increase and found that hyPE2 and PE2-mid_RPA70 showed a median of 1.6-fold (range, 1.1- to 11-fold) and a median of 1.2-fold (range, 0.57- to 6.6-fold) higher efficiency than PE2 (Fig. 1b). In addition, regarding the 34 pairs that showed a PE2 efficiency between 0% and 1%, 15 and 13 pairs showed a prime-editing efficiency higher than 1% with hyPE2 (an average efficiency of 11%, a median efficiency of 9.1%, ranging from 1.1% to 29%) and PE2-mid_RPA70 (an average efficiency of 7.4%, a median efficiency of 5.3%, ranging from 1.1% to 24%), respectively. Of the 19 pairs associated with 0% PE2 efficiencies, 3 and 2 pairs showed an efficiency higher than 1% with hyPE2 (1.8, 2.3, and 4.0%) and PE2-mid_RPA70 (1.2%, 1.5%), respectively. Based on these results, we used Rad51 DBD for subsequent studies.

We also inserted Rad51 DBD into the N- or C-terminal regions of PE2, generating variants named PE2-N_Rad51 and PE2-C_Rad51, respectively (Fig. 1a). When we tested these two variants together with hyPE2 in comparison with PE2 at target sequences that showed a PE2 efficiency higher than 1%, hyPE2 showed the highest overall activities (median, 1.8-fold higher than PE2 activities), whereas PE2-N_Rad51 revealed lower activities than PE2 (median, 0.85-fold) and PE2-C_Rad51 showed slightly higher overall activities than PE2 (median, 1.1-fold) (Fig. 1c). These higher hyPE2 efficiencies compared with those of PE2 were observed at all 33 tested target sequences that showed PE2-driven prime-editing efficiencies higher than 1%. Among the remaining 55 target sequences with PE2-driven prime-editing efficiencies lower than 1%, 20 target sequences (20/55 = 36%) showed hyPE2-induced prime-editing efficiencies higher than 1%, with an average efficiency of 9.1% (median 5.7%, range, 1.1–29%) (Fig. 1d). These experiments support the use of hyPE2 in later experiments.

To test hyPE2 in a different cell line, we generated a library of HCT116 cells containing the 107 pairs of pegRNA-encoding and target sequences. Similar to previous analyses, 26 pairs with insufficient reads (<100 reads) and 38 pairs with PE2 efficiency <1% were excluded. The evaluation of prime-editing efficiencies for the remaining 43 pairs showed that hyPE2 had a median of 1.4-fold (range, 0.82- to 2.9-fold) higher efficiency than PE2 (Fig. 1e).

**Fig. 2:**
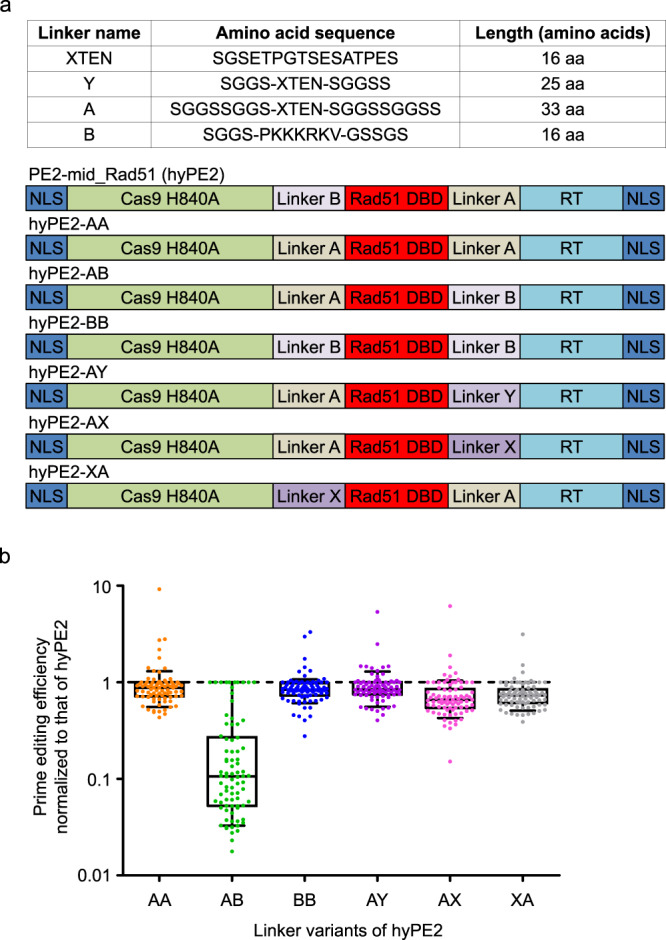
Evaluation of hyPE2 linker variants. **a** Detailed information about the linkers that were used and the structures of the hyPE2 linker variants. DBD DNA-binding domain, NLS nuclear-localization signal, RT reverse transcriptase. **b** Prime-editing efficiencies of hyPE2 variants normalized to the efficiency of hyPE2 at the same target sequences, which had been lentivirally integrated in HEK293T cells. Adjusted fold increases are shown on the y axis. Data of minimum-to-maximum values are presented. For the boxes, the top, middle, and bottom lines represent the 25th, 50th, and 75th percentiles, respectively. The whiskers indicate the 10th- and 90th-percentile values. The number of pegRNAs *n* = 82. Source data are provided as a Source Data file.

Next, to evaluate the effects of different linkers on the activity of hyPE2, we prepared six linker variants of hyPE2, named hyPE2-AA, -AB, -BB, -AY, -AX, and -XA (Fig. 2a). When we evaluated the activities of these variants in comparison with that of hyPE2 using cell library A described above, all of these variants showed lower prime-editing efficiencies than hyPE2 (Fig. 2b). Thus, we used hyPE2 for subsequent studies.

### Testing hyPE2 at endogenous targets

**Fig. 3:**
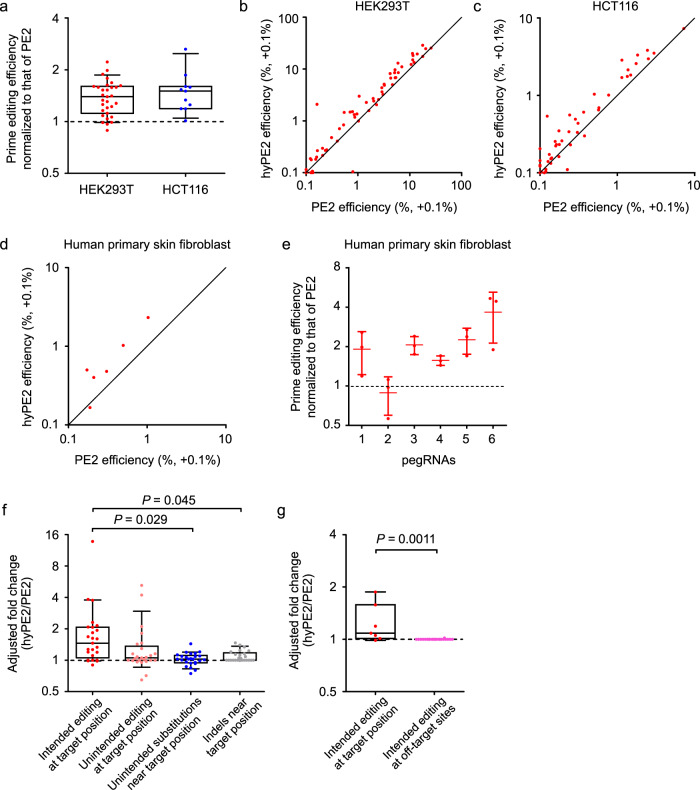
hyPE2 has higher prime-editing efficiencies than PE2 at endogenous target sites. **a** Prime-editing efficiencies of hyPE2 normalized to the efficiency of PE2 at the same endogenous targets in HEK293T cells and HCT116 cells. PegRNAs that resulted in PE2-directed prime-editing efficiencies higher than 1% are shown; the number of pegRNAs *n* = 31 for HEK293T cells and *n* = 11 for HCT116 cells. **b**, **c** Comparison of the prime-editing efficiencies of PE2 and hyPE2 at endogenous sites in HEK293T cells (**b**) and HCT116 cells (**c**). The number of pegRNAs *n* = 63 (b) and *n* = 51 (**c**). **d** Comparison of the prime-editing efficiencies of PE2 and hyPE2 at 6 endogenous sites in primary human skin fibroblasts. **e** Prime-editing efficiencies of hyPE2 normalized to the efficiency of PE2 at the same target sequences in primary human skin fibroblasts. Adjusted fold increases are shown on the y axis. Data are means ± S.D. for three independent biological replicates. **f** Prime editing and unintended editing frequencies of hyPE2 normalized to those of PE2 at the same endogenous targets in HEK293T cells. Adjusted *p*-values calculated by one-way ANOVA with post hoc analysis by Tukey’s multiple comparisons are shown. For brevity, we have not included *p*-values when the differences between the values of the two groups are not statistically significant. The number of pegRNAs *n* = 25. **g** On-target and off-target editing frequencies of hyPE2 normalized to those of PE2 at the same endogenous targets in HEK293T cells. The *p*-value was calculated by two-tailed, unpaired Student’s *t*-test. The number of pegRNAs *n* = 7 (on-target) and the number of pegRNA off-target pairs *n* = 22. **a**, **f**, **g** Data of minimum-to-maximum values are presented. For the boxes, the top, middle, and bottom lines represent the 25th, 50th, and 75th percentiles, respectively. The whiskers indicate the 10th- and 90th-percentile values. **b**–**d** The black line indicates *y* = *x*. The data points represent the average prime editing efficiency from three biological replicates at each target sequence. 0.1% was added﻿ to all efficiency values so that log scales could be used for both the x- and y axes. Source data are provided as a Source Data file.

We then compared the efficiencies of hyPE2 and PE2 at 63 and 51 endogenous target sites (Supplementary Table 1) in HEK293T and HCT116 cells, respectively. PE2 efficiencies were higher than 1% at 31 and 11 targets in HEK293T and HCT116 cells, respectively; at these sites, the efficiencies of hyPE2 were a median of 1.4-fold (range, 0.89- to 2.2-fold) and 1.5-fold (range, 1.0- to 2.6-fold) higher than those of PE2 in HEK293T and HCT116 cells, respectively (Fig. 3a–c). Notable hyPE2-directed increases in the prime-editing efficiencies were from 5.8% to 13% in HEK293T cells and from 1.1% to 2.8% in HCT116 cells. At the remaining 32 and 40 target sites where PE2 efficiencies were lower than 1%, 11 target sequences (34% = 11/32) showed hyPE2-induced prime-editing efficiencies higher than 1%, with an average efficiency of 1.6% (a median of 1.4%, range, 1.1–2.9%) in HEK293T cells, whereas hyPE2 efficiencies were also all lower than 1% in HCT116 cells. These results corroborate that hyPE2 has an overall higher activity than PE2.

We also compared the efficiencies of hyPE2 with those of PE2 in primary human skin fibroblasts, a therapeutically relevant cell type, at six target sequences. HyPE2 showed higher prime-editing efficiencies at five out of the six targets and the mean- and median-fold increases at the six targets were 2.1- and 2.0-fold (adjusted fold, Methods), respectively (Fig. 3d, e).

### Unintended editing and off-target effects generated by hyPE2

We next determined whether hyPE2 induces a higher level of unintended edits than PE2 at the endogenous target sequences. To compare the frequencies of unintended edits, we adopted adjusted fold increases (Methods). The levels of unintended substitutions and edits such as indels slightly increased when hyPE2 was used instead of PE2, although the fold increases in these unintended edits were lower than or at most comparable to those for the intended edits (Fig. 3f).

**Fig. 4:**
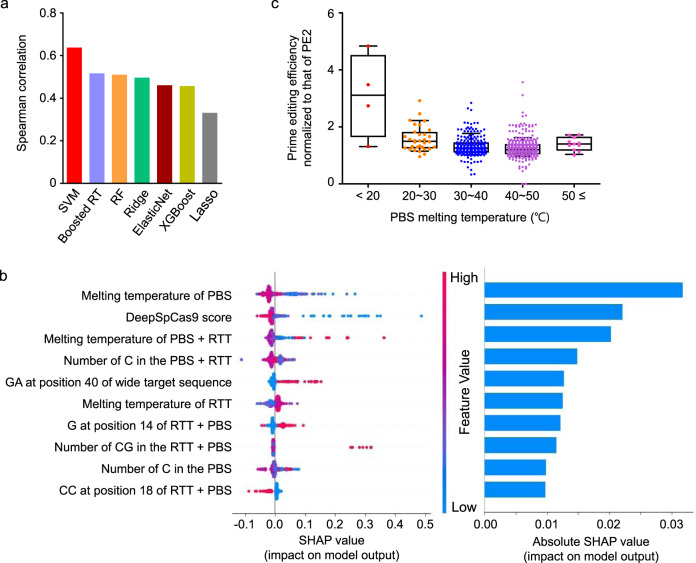
Development and evaluation of PEselector, a computational model that predicts the fold increase of hyPE2-induced prime-editing efficiencies as compared to those of PE2. **a** Comparison of Spearman correlation coefficients between prediction models. SVM, support-vector machine; Boosted RT, gradient-boosted regression tree; RF, random forest; Ridge, L2-regularized linear regression; ElasticNet, L1L2-regularized linear regression; XGBoost, extreme-gradient boosting; Lasso, L1-regularized linear regression. **b** The top ten features associated with hyPE2 activity as compared with PE2 activity determined by Tree SHAP (XGBoost classifier). The dot colors indicate the high (red) or low (blue) values of the relevant feature for each pegRNA. Overlapping points are slightly separated in the y-axis direction, so that density is apparent. **c** Dependence of the fold increase in hyPE2 prime-editing efficiency relative to that of PE2 on the primer-binding site (PBS) melting temperature. Editing efficiencies for hyPE2 and PE2 were determined at the same target sequences, which had been lentivirally integrated in HEK293T cells (library B). Data points are overlaid. The number of pegRNAs *n* = 4 (<20 °C), 32 (20–30 °C), 236 (30–40 °C), 348 (40–50 °C), and 11 (≥50 °C). Data of minimum-to-maximum values are presented. For the boxes, the top, middle, and bottom lines represent the 25th, 50th, and 75th percentiles, respectively. The whiskers indicate the 10th- and 90th-percentile values. Source data are provided as a Source Data file.

We also evaluated off-target effects by analyzing potential off-target sites of pegRNAs that showed relatively high on-target prime-editing efficiencies in HEK293T cells. We first identified potential off-target sites that have up to two nucleotide mismatches or a one-nucleotide RNA or DNA bulge per pegRNA and found a total of six potential off-target sites for three pegRNAs (Supplementary Table 2, Supplementary Fig. 3). We also determined prime-editing efficiencies using four *HEK4*-targeting pegRNAs, which were associated with a total of 16 pairs of pegRNAs and off-target sites in the initial study of prime editing^1^ (Supplementary Table 2, Supplementary Fig. 3). An evaluation of these 22 (= 6 + 16) pairs of pegRNAs and potential off-target sites in HEK293T cells showed that the off-target effects of hyPE2 were comparable to those of PE2 (Fig. 4g, Supplementary Fig. 3).

### Development of PEselector

Given that the hyPE2-induced increases in prime-editing efficiencies were higher for some pegRNAs than others, we next attempted to predict hyPE2-directed fold increases in prime-editing efficiencies. For this purpose, we compared hyPE2- and PE2-induced prime-editing efficiencies using 665 pairs of pegRNAs and integrated target sequences (library B) using the high-throughput method described above. When we combined the fold-increase data from libraries A and B, the mean- and median-fold increases were 1.3- and 1.2-fold (Supplementary Fig. 4). The data from library B were randomly split into training and test datasets such that neither pegRNAs nor target sequences are shared between the two datasets (Supplementary Table 3). Using the training dataset of hyPE2- and PE2-induced prime-editing efficiencies determined with 568 pegRNAs, we generated seven computational models that predict the fold increase of hyPE2-induced prime-editing efficiencies as compared to those of PE2 and found that a support-vector machine-based model showed the highest performance (Fig. 4a). We named the model PEselector and provide it as a web tool at http://deepcrispr.info/PEselector.

### Factors associated with hyPE2 efficiency

To identify the factors associated with the fold increases in hyPE2-induced prime-editing efficiencies, we performed XGBoost combined with SHAP^14^ and found that the most important factor out of the 1820 features (Methods) was the melting temperature of the primer-binding site (PBS) (Fig. 4b). When we checked how the fold increase in the hyPE2 efficiency depended on the melting temperature of the PBS, we found that the fold increase tended to decrease as the PBS melting temperature increased (Fig. 4c), suggesting that hyPE2 will be especially useful when the PBS melting temperature is low.

To find potential mechanisms underlying the higher prime editing efficiency of hyPE2, we predicted the three-dimensional structures of PE2 and hyPE2 (Supplementary Fig. 5). Given that the Rad51 DBD can promote the formation of DNA/RNA hybrids by binding to both ssDNA and RNA^15^, Rad51 may facilitate the binding of the pegRNA to the nicked target ssDNA, enhancing reverse transcription. This potential mechanism is in line with the finding that the enhancement of prime-editing efficiencies is especially high when the PBS melting temperature is low, which could be linked with poor binding of the pegRNA PBS domain to the nicked target ssDNA when PE2 is used. However, the elucidation of the exact mechanism underlying the enhanced activity of hyPE2 would require an additional study.

### Concluding remarks

Taken together, our results indicate that hyPE2, which contains a Rad51 DBD, can show higher prime-editing efficiencies than PE2 at lentivirally integrated and endogenous target sequences in HEK293T cells, HCT116 cells, and primary human skin fibroblasts. PEselector is provided as a webtool, so that users can identify pegRNAs that are expected to be especially efficient when combined with hyPE2 as compared with PE2. We envision that hyPE2 will facilitate the biomedical and biotechnological applications of prime editing.

## Methods

### Plasmid vectors

Sequences encoding human RPA70-C and Rad51DBD were synthesized by GeneScript, after which they were amplified by PCR and cloned into the pCMV-PE2 (Addgene, no. 132775) plasmid to generate the ssDBD-PE2-encoding plasmids. These plasmids were named PE2-mid_RPA70, hyPE2, PE2-N_Rad51, and PE2-C_Rad51 (Fig. 1a). Linker variants were derived from the hyPE2 plasmid and cloned using Gibson assembly^16^. The sequences of the primers and plasmids used in this study are shown in Supplementary Table 4 and the Supplementary Note, respectively.

### Plasmid-library preparation and cell-library generation

We previously generated a plasmid library of 54,836 pairs of pegRNA-encoding and target sequences^2^. We randomly selected 107 plasmids from this library by colony picking and mixed the selected plasmids at an equimolar ratio (library A).

Next, we additionally designed another library named library B that includes 665 pairs of pegRNAs and target sequences for a more extensive evaluation of various types of editing at a larger number of target sequences. To design library B, we selected 100 deletion-, 100 insertion-, and 200 substitution-inducing pegRNAs from the previously published library of 54,836 pairs of pegRNA-encoding and target sequences^2^. For this selection, we divided the editing efficiencies from the previous study^2^ into eight strata (<1%, 1–3%, 3–6%, 6–10%, 10–20%, 20–30%, 30–40%, and >40%) and randomly selected a similar number of pegRNAs from each stratum, so that pegRNAs associated with all levels of efficiency would be included. To these 400 pegRNAs, we added 107 pegRNAs used in library A, resulting in a total of 507 pegRNAs. Among the 507 pegRNAs, 158 could be modified to induce a silent mutation in the NGG PAM sequence; we added the 158 modified pegRNAs, which can induce a silent mutation in the PAM sequence in addition to the initially designed edit, to library B. Thus, the total number of pegRNAs in library B was 507 + 158 = 665. Each pegRNA was associated with three barcodes. Thus, the number of oligonucleotides used to generate library B was 665 × 3 = 1995.

In preparation for generating lentivirus from the library of 107 plasmids, HEK293T cells were seeded at a density of 4.0 × 10^6^ cells per plate on 100-mm dishes that contained Dulbecco’s Modified Eagle Medium (DMEM). After 15 h, the culture medium was replaced with DMEM containing 25 μM chloroquine diphosphate (Sigma) and the cells were incubated for another 5 h. The plasmid library was mixed with psPAX2 (Addgene no. 12260) and pMD2.G (Addgene no. 12259) at a molar ratio of 1.3:0.72:1.64; the plasmids were then cotransfected into HEK293T cells using polyethylenimine (PEI MAX, Polysciences). The next day, the culture medium was replaced with fresh medium. At 48 hrs after the transfection, the medium, which contained the lentivirus, was collected and filtered using a Millex-HV 0.45-μm low protein-binding membrane (Millipore). The filtrate was then aliquoted and stored at −80 °C. For titration of the lentivirus, serial dilutions of a viral aliquot were transduced, in the presence of 8 μg/ml polybrene (Sigma), into HEK293T cells that had been cultured in DMEM supplemented with 10% fetal bovine serum (FBS). Untransduced and transduced cells were then both cultured in DMEM supplemented with 10% FBS and 2 μg/ml of puromycin (Invitrogen). After essentially all of the untransduced cells had died, we counted the number of living cells in the transduced population to estimate the viral titer^17^.

For lentivirus transduction, HEK293T or HCT116 cells were seeded on 100-mm dishes at a density of 1.0 × 10^6^ cells per dish and incubated overnight. The lentiviral library was transduced at an MOI of 0.3 to achieve a coverage greater than 3000 × relative to the number of selected pegRNA-encoding plasmids. The next day, the culture medium was replaced with DMEM supplemented with 10% FBS and 2 μg/ml puromycin (InvivoGen). Cultures were maintained with these conditions for the next five days to remove untransduced cells.

### Delivery of PE2 or PE2 variants into the cell library

To deliver each PE2 variant to cell library A or B, PE2 variant-, pcDNA-BSD-, and puro-eGFP-encoding plasmids were mixed at a weight ratio of 10:1:1 to yield a total of 12 μg (for experiments using library 1) or 24 μg (for library B) of plasmid mixture, which was then transfected into a total of 1 × 10^6^ cells from cell library A or a total of 6 × 10^6^ cells from cell library B using Lipofectamine 2000 (Invitrogen), following the manufacturer’s protocol. After incubation overnight, the culture medium was exchanged with DMEM containing 10% FBS and 40 μg/ml blasticidin S (InvivoGen). Five days later, the transfected cells were harvested with 0.25% trypsin for genomic DNA extraction and deep sequencing.

### Measurement of prime-editing activities at endogenous sites

To evaluate hyPE2 and PE2 activities at endogenous sites, HEK293T or HCT116 cells were seeded into 24-well plates and transfected at 70–80% confluency. In all, 750 ng of PE2-, 250 ng of pegRNA-, and 100 ng of eGFP-Puro- (Addgene no. 45561) encoding plasmids were mixed and co-transfected into the cells using Lipofectamine 2000, following the manufacturer’s protocol. The next day, the culture medium was replaced with DMEM supplemented with 10% FBS and 2 μg/ml puromycin (InvivoGen). Five days later, the transfected cells were harvested with 0.25% trypsin for genomic DNA extraction and deep sequencing.

After written informed consent was obtained from a study participant who is a healthy individual, a dermatology specialist conducted skin-punch biopsy from the participant. The Institutional Review Board of Severance Hospital, Yonsei University Health System approved the consent procedure and the study (No. 4-2012-0028). The fibroblasts derived from the skin biopsy were cultured in DMEM containing 10% FBS and penicillin/streptomycin. A total of 1 × 10^6^ human skin fibroblasts were mixed with 3 μg of PE2-, 1 μg of pegRNA-, and 1 μg of eGFP-Puro-encoding plasmids and electroporated using a Neon electroporation kit, following the manufacturer’s protocol. Five days after the transfection, the cells were harvested with 0.25% trypsin for genomic DNA extraction and deep sequencing.

### Deep sequencing

The protocol used for deep sequencing has been previously described^2,18–20^. Briefly, genomic DNA was extracted from pelleted cells using a Wizard Genomic DNA purification kit (Promega), following the manufacturer’s protocol. To measure prime editing efficiencies for the library experiments, a total of 16 μg (greater than 16,000 × coverage) of genomic DNA was PCR-amplified using a 2× pfu PCR Smart mix (Solgent). The resulting PCR products were combined and purified with a MEGAquick-spin total fragment DNA purification kit (iNtRON Biotechnology). Next, 20 ng of purified product was PCR-amplified using primers containing Illumina adapter and barcode sequences. To determine prime-editing efficiencies at endogenous sites, ~200 ng of individual genomic DNA samples were PCR-amplified in 20-μl reaction volumes. The resulting PCR products were combined and purified. Next, 100 ng of purified product was PCR-amplified in a 20 μl reaction volume using primers containing Illumina adapter sequences. The resulting products were purified and sequenced with MiniSeq (Illumina). The primers used for PCRs are listed in Supplementary Table 4.

### Analysis of prime-editing activities

The prime-editing efficiencies (i.e., the frequencies of intended edits) in the library experiments were calculated using previously published Python scripts^2^ as follows:

1 $$\frac{{{{{{\rm{Read}}}}}}\,{{{{{\rm{counts}}}}}}\,{{{{{\rm{with}}}}}}\,{{{{{\rm{intended}}}}}}\,{{{{{\rm{edit}}}}}}\,{{{{{\rm{and}}}}}}\,{{{{{\rm{specified}}}}}}\,{{{{{\rm{barcode}}}}}}}{{{{{{\rm{Total}}}}}}\,{{{{{\rm{read}}}}}}\,{{{{{\rm{counts}}}}}}\,{{{{{\rm{with}}}}}}\,{{{{{\rm{specified}}}}}}\,{{{{{\rm{barcode}}}}}}}{{\times}}100$$

To identify individual pegRNA and target–sequence pairs, a 22-nt sequence, consisting of an 18-nt barcode and a 4-nt sequence upstream of the barcode, was used. To improve the accuracy of our analysis, pegRNA and target-sequence pairs with deep-sequencing read counts below 100 were excluded^2,19,21^. The reads that contained the desired edit but lacked unintended mutations in the wide target sequence containing the PAM were classified as PE2-induced mutations.

To evaluate the frequencies of intended edits, unintended edits, and indels at endogenous sites, Cas-analyzer was used^22^ and the values were calculated as described below. For analysis of unintended substitutions near the target position, a 40-nt region spanning from -10 nucleotides (nts) to +25 nts from the nick site was evaluated for substitutions and the average values were considered as read counts for subsequent calculations.

2 $${{{{{\rm{Intended}}}}}}\,{{{{{\rm{editing}}}}}}\,{{{{{\rm{frequency}}}}}}=\frac{{{{{{\rm{Read}}}}}}\,{{{{{\rm{counts}}}}}}\,{{{{{\rm{with}}}}}}\,{{{{{\rm{intended}}}}}}\,{{{{{\rm{edit}}}}}}}{{{{{{\rm{Total}}}}}}\,{{{{{\rm{read}}}}}}\,{{{{{\rm{counts}}}}}}}{{\times}}100$$

3 $${{{{{\rm{Unintended}}}}}}\,{{{{{\rm{editing}}}}}}\,{{{{{\rm{frequency}}}}}}=\frac{{{{{{\rm{Read}}}}}}\,{{{{{\rm{counts}}}}}}\,{{{{{\rm{with}}}}}}\,{{{{{\rm{unintended}}}}}}\,{{{{{\rm{edit}}}}}}}{{{{{{\rm{Total}}}}}}\,{{{{{\rm{read}}}}}}\,{{{{{\rm{counts}}}}}}}{{\times}}100$$

4 $${{{{{\rm{Indel}}}}}}\,{{{{{\rm{frequency}}}}}}=\frac{{{{{{\rm{Read}}}}}}\,{{{{{\rm{counts}}}}}}\,{{{{{\rm{with}}}}}}\,{{{{{\rm{indel}}}}}}}{{{{{{\rm{Total}}}}}}\,{{{{{\rm{read}}}}}}\,{{{{{\rm{counts}}}}}}}{{\times}}100$$

In some cases, we calculated an adjusted fold increase in which +0.1% was added to both the hyPE2 and PE2 efficiencies in order to avoid mathematical errors that would have otherwise been generated when the PE2 efficiency is 0% and to attenuate insignificant fold increases as shown below.

5 $${{{{{\rm{Adjusted}}}}}}\,{{{{{\rm{fold}}}}}}\,{{{{{\rm{change}}}}}}=\frac{{{{{{\rm{hyPE}}}}}}2\,{{{{{\rm{efficiency}}}}}}\,( \% )+0.1 \% }{{{{{{\rm{PE}}}}}}2\,{{{{{\rm{efficiency}}}}}}\,( \% )+0.1 \% }$$

For example, the adjusted fold increase from 0.015% to 0.15% can be calculated as (0.15% + 0.1%)/(0.015% + 0.1%) = 2.2-fold instead of 10-fold; however, the increase from 1.5% to 15% can be calculated as (15% + 0.1%)/(1.5% + 0.1%) = 9.4-fold, which is close to 10-fold. When we used an adjusted fold increase instead of the fold increase, we mention this point in the legends to the relevant figures.

### Measurement of prime-editing activities at potential PE2 off-target sites

Potential PE2 off-target sites that have up to two nucleotide mismatches or a one-nucleotide RNA or DNA bulge were identified by Cas-OFFinder^23^. Information about the potential off-target sites is shown in Supplementary Table 2. To evaluate prime-editing efficiencies at the potential off-target sites, the genomic DNA samples that were used for the measurement of prime-editing activities at endogenous sites described above were used as templates for PCR amplification. The resulting products were purified and sequenced with MiSeq.

### Conventional machine learning-based model training

The data of hyPE2- and PE-induced prime-editing efficiencies obtained using library B were split into training and test datasets by random sampling, such that neither pegRNAs nor target sequences are shared between the two datasets (Supplementary Table 3). Each of seven conventional machine learning algorithms—extreme-gradient boosting (XGBoost), gradient-boosted regression tree (Boosted RT), random forest, L1-regularized linear regression (Lasso), L2-regularized linear regression (Ridge), L1L2-regularized linear regression (ElasticNet) and support-vector machine (SVM)—were used to train a model. We used the XGBoost Python package (version 1.3.3)^24^ and scikit-learn (version 0.23.2)^25^. A set of 1820 features, including position-independent and position-dependent nucleotides and dinucleotides, melting temperature, GC counts, the minimum self-folding free energy^26,27^, and the DeepSpCas9 score^27^, were extracted from the wide target sequences and the PBS and RT-template sequences^2^. The MeltingTemp module (https://biopython.org/docs/1.74/api/Bio.SeqUtils.MeltingTemp.html) was used to calculate the melting temperature using a default setting. To select a model from the regularization parameters and hyperparameter configurations in each algorithm, fivefold cross-validation was done. Details for each of the machine-learning algorithms follow. XGBoost and gradient-boosted regression tree: we searched over 16 models that had been chosen from various hyperparameter configurations {the number of base estimators (chosen from [50, 100]), the maximum depth of the individual regression estimators (chosen from [5, 10]), the minimum number of samples to be at a leaf node (chosen from [1, 2]), and learning rate (chosen from [0.1, 0.2])}. Random forest: we searched over 16 models chosen from the same hyperparameter configurations used for XGBoost, except that the learning rate was not used; we searched over the maximum number of features to consider when looking for the best split (chosen from [all features, the square root of all features, the binary logarithm of all features]). L1-, L2-, and L1L2-regularized linear regression: we searched over 16 points that were evenly spaced between 10^−6^ and 10^6^ in log space to optimize the regularization parameter. SVM: we searched over 16 models from the following hyperparameters: penalty parameter C and kernel parameter *γ*, four points that were evenly spaced between 10^−3^ and 10^3^.

### Three-dimensional structural modeling

The structural model for hyPE2 shown in Supplementary Fig. 5b was built with the Coot program (version WinCoot 0.9.6.1)^28^. The three-dimensional model of Cas9 in complex with a guide RNA and a target DNA fragment was obtained from the structure of a SpCas9 DNA adenine-base editor (PDB code: 6VPC)^29^. To model the 3′ extension of a 121-nt pegRNA (residues 83–121) manually, we used RNAfold WebServer^30^ to predict the secondary structure of this region and adopted a hairpin structure for residues 83–97. The pegRNA RT-template region hybridized with the 16-nt DNA primer region was manually modeled based on the structure of XMRV RT in complex with an RNA:DNA hybrid (PDB code: 4HKQ)^31^. The three-dimensional model of the Rad51 ssDBD (residues 16–85) was obtained from the structure of the N-terminal domain of Rad51 (PDB code: 1B22)^32^. To find putative α-helices in flexible N- and C-terminal regions of the Rad51 ssDBD, Linker A, and Linker B, we predicted the secondary structures using RaptorX^33^. Figures showing the three-dimensional structures (Supplementary Fig. 5b) were produced using the UCSF Chimera program^34^, and two linkers were represented on the three-dimensional structures, taking into account their lengths and secondary structures.

For the schematic structural model of hyPE2 shown in Supplementary Fig. 5a, we used the coordinates of Cas9 (PDB 4OO8), RT (PDB 5DMQ), and Rad51 (PDB 1B22). The structural image was prepared using the program CueMol (version 2.2.3.443; http://www.cuemol.org).

### Statistics and reproducibility

Data are presented as means ± S.D. from independent experiments. *P*-values were calculated by two-tailed, unpaired Student’s *t*-test or one-way ANOVA with post hoc analysis by Tukey’s multiple comparisons, depending on the number of independent variables. The high-throughput experiments were independently repeated three times for library A, and two times for library B and linker variants. All replications showed similar results. The individual evaluation experiments of HEK293T cells, HCT116 cells, and human fibroblasts were independently repeated three times, with comparable results.

### Reporting summary

Further information on research design is available in the Nature Research Reporting Summary linked to this article.

## Supplementary information

Supplementary Information

Reporting Summary

Peer Review File

## Data Availability

Source data are provided with this paper. The deep-sequencing data from this study have been submitted to the National Center for Biotechnology Information Sequence Read Archive under accession number SRP307854. The protein-structure data for predicting the structure of PE2 and hyPE2 are from Protein Data Bank (https://www.rcsb.org). PDB code is 6VPC for SpCas9 DNA adenine-base editor (10.2210/pdb6VPC/pdb), 4HKQ for XMRV RT in complex with an RNA:DNA hybrid (10.2210/pdb4HKQ/pdb), 1B22 for N-terminal domain of Rad51 (10.2210/pdb1B22/pdb), 4OO8 for Cas9 (10.2210/pdb4OO8/pdb), and 5DMQ for reverse transcriptase (10.2210/pdb5DMQ/pdb), respectively. Source data are provided with this paper.

## Source data

Source Data

## Code availability

The Python script used for prime-editing efficiency calculations has been published^2^ and is available at https://github.com/hkimlab-PE/PE_SupplementaryCode.
