# Supplementary material for: Generation of a more efficient prime editor 2 by addition of the Rad51 DNA-binding domain
Source: Nat Commun. 2021 Sep 23;12:5617. doi: 10.1038/s41467-021-25928-2 (PMC8460726; doi:10.1038/s41467-021-25928-2)
Supplement: Supplementary file 1 — Supplementary Information [file 41467_2021_25928_MOESM1_ESM.pdf]

Supplementary Information

Generation of a more efficient prime editor 2 by addition of the Rad51 DNA-binding domain

Contents:

Supplementary Figures 1 – 5
Supplementary Tables 1 – 4
Supplementary Note

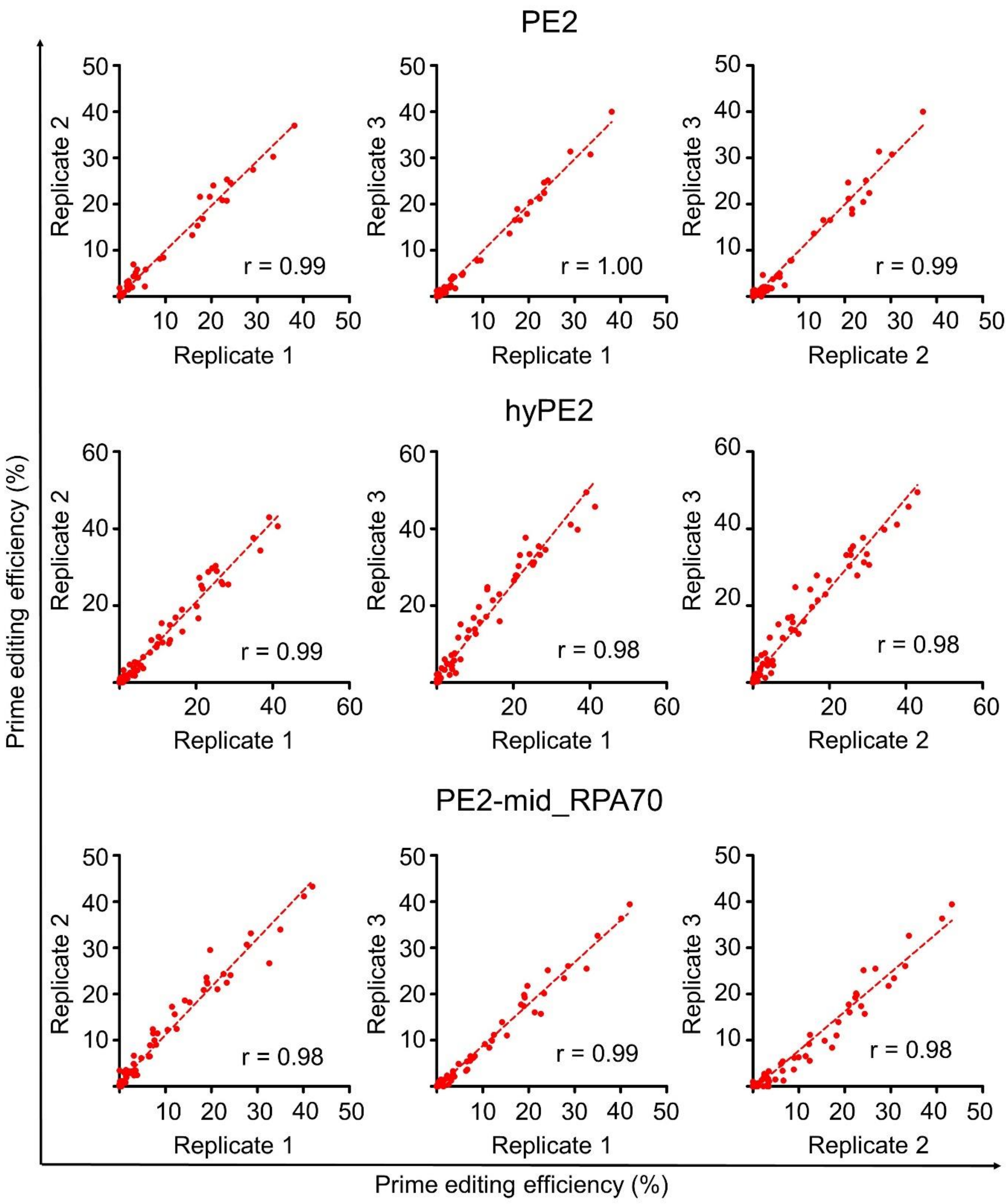

Supplementary Figure 1. Correlations between prime editing efficiencies from biological replicates for high-throughput evaluations of prime editing activities. The biological replicates were independently cultured, transfected with plasmids encoding prime editor 2 (variants), and analyzed. The Pearson correlation coefficients (r) and trend lines are shown. The number of analyzed target sequences *n* = 83. Source data are provided as a Source Data file.

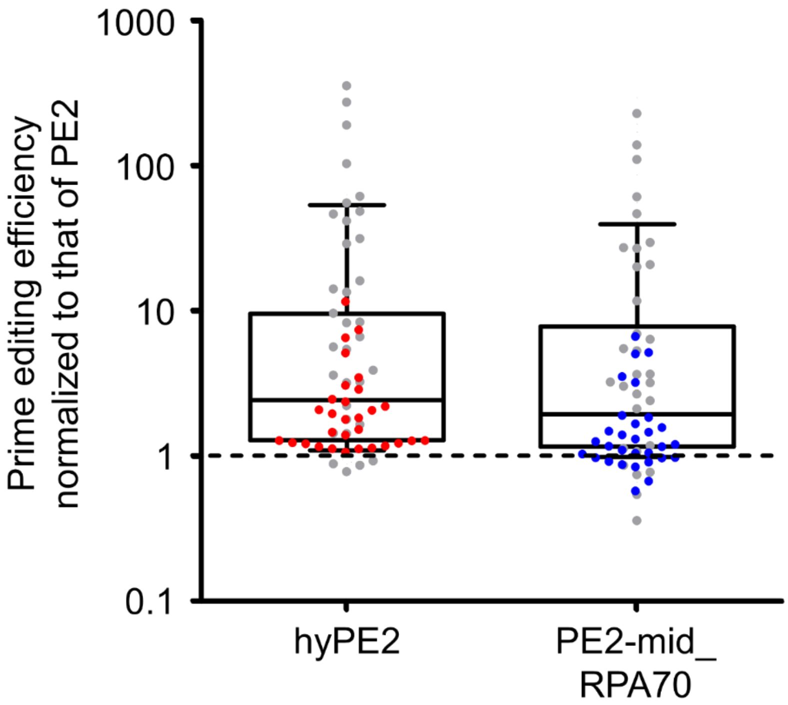

Supplementary Figure 2. Prime editing efficiencies of PE2 variants normalized to the efficiency of PE2 at the same target sequences, which had been lentivirally integrated in HEK293T cells. Target sequences with PE2-induced prime editing efficiencies less than 1% are shown as gray dots. The number of target sequences *n* = 64. Data of minimum to maximum values are presented. For the boxes, the top, middle, and bottom lines represent the 25th, 50th, and 75th percentiles, respectively. The whiskers indicate the 10th and 90th percentile values. Source data are provided as a Source Data file.

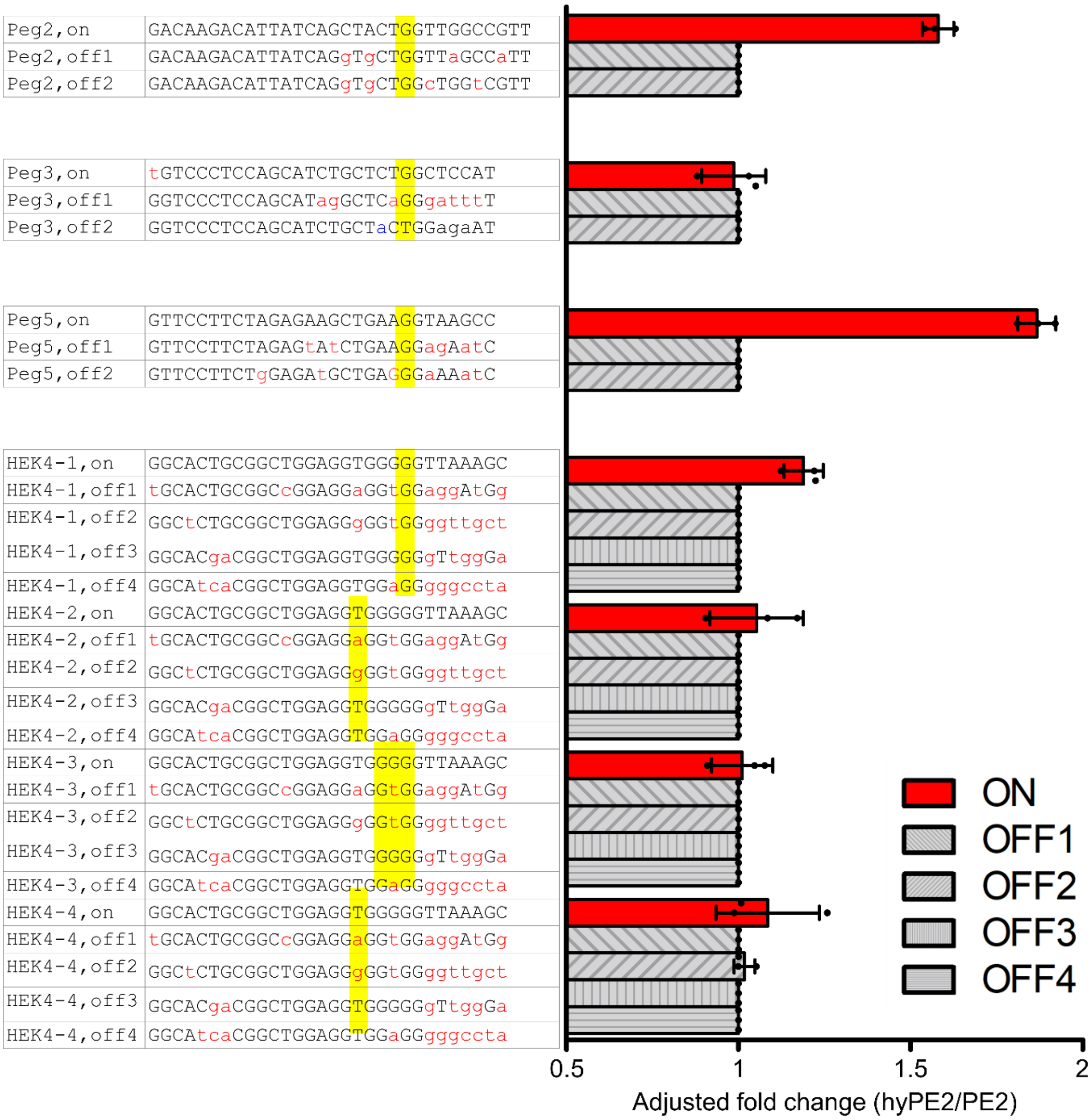

Supplementary Figure 3. Off-target effects of hyPE2 and PE2 at the potential off-target sites for pegRNAs 2, 3, and 5 and for four different pegRNAs targeting HEK4 in HEK293T cells. The intended editing positions are highlighted in yellow; mismatched and bulged nucleotides are indicated in red and blue lower-case fonts, respectively. Data points are overlaid. Data are means ± S.D. for three independent biological replicates. on, on-target site; off, off-target site. Source data are provided as a Source Data file.

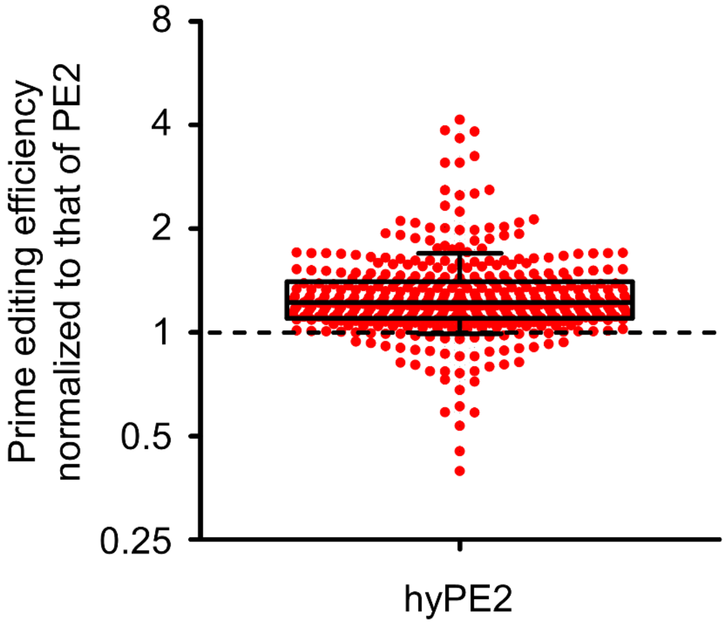

Supplementary Figure 4. Prime editing efficiencies of PE2 variants normalized to the efficiency of PE2 at the same target sequences, which had been lentivirally integrated in HEK293T cells. The number of target sequences *n* = 423. PegRNAs that resulted in PE2-directed prime editing efficiencies higher than 1% are shown. Data of minimum to maximum values are presented. For the boxes, the top, middle, and bottom lines represent the 25th, 50th, and 75th percentiles, respectively. The whiskers indicate the 10th and 90th percentile values. Source data are provided as a Source Data file.

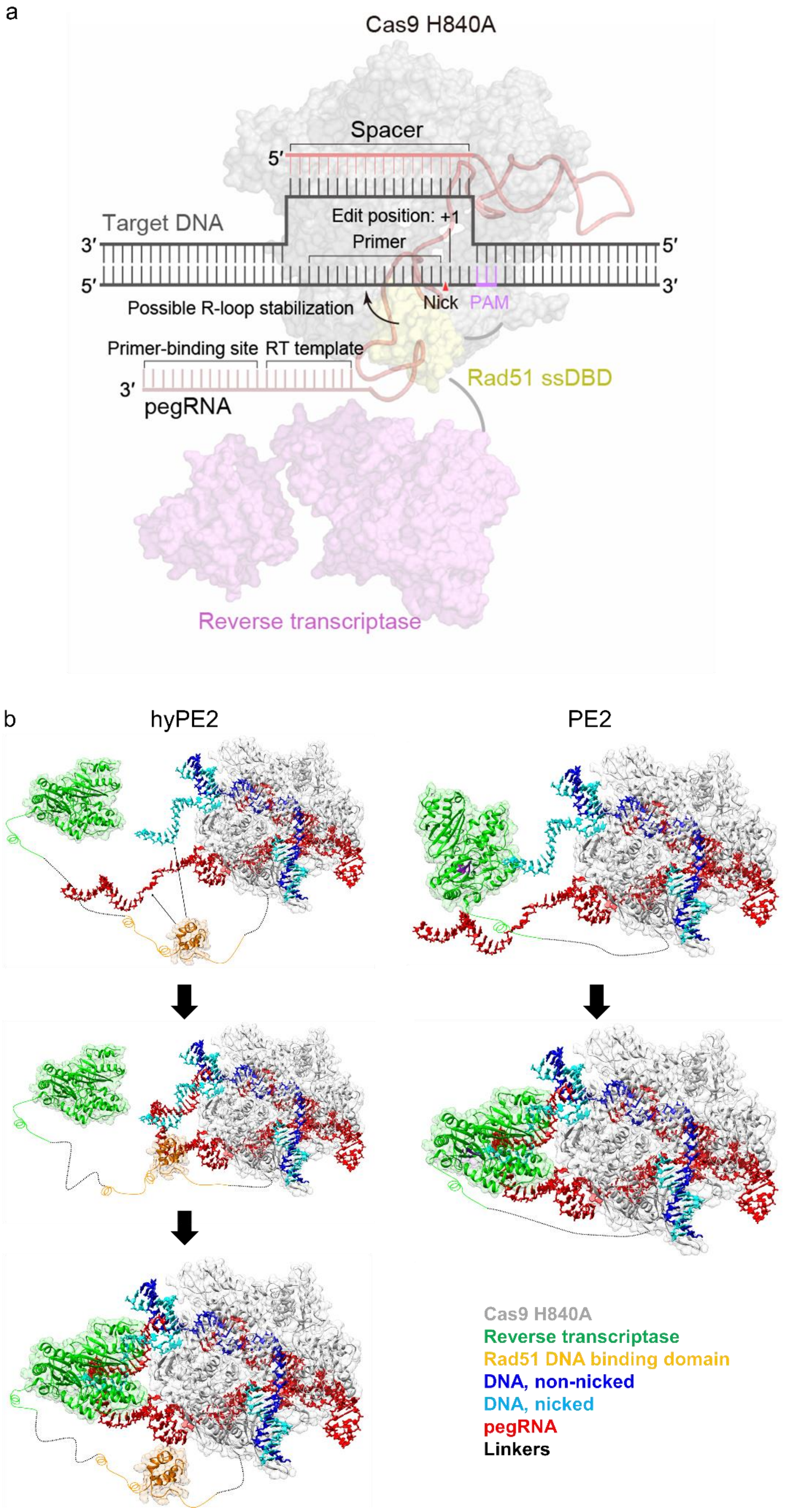
a
Cas9 H840A
Spacer
5′
Target DNA
Edit position: +1
Primer
3′
5′
5′
3′
Possible R-loop stabilization
Nick
PAM
Primer-binding site
RT template
3′
pegRNA
Rad51 ssDBD
Reverse transcriptase
b
hyPE2
PE2
Cas9 H840A
Reverse transcriptase
Rad51 DNA binding domain
DNA, non-nicked
DNA, nicked
pegRNA
Linkers

Supplementary Figure 5. Structure of hyPE2. **a,** Schematic representation of hyPE2. **b,** Three-dimensional structures predicted for hyPE2 (left) and PE2 (right) before and after binding of the reverse transcriptase domain to the nicked target ssDNA/pegRNA hybrid. Modeling of a hypothetical interaction between Rad51, the nicked target ssDNA, and the pegRNA primer binding site is shown for hyPE2. Gray, Cas9 H840A; Green, Reverse transcriptase; Yellow, Rad51 DNA binding domain; Blue, DNA, non-nicked; Light blue, DNA, nicked; Red, pegRNA; Black, Linkers.

Supplementary Table 1. Characteristics (sequences of target, pegRNA spacer, and extension) of pegRNAs used in this study and relevant editing efficiencies (provided as a separate file).

Supplementary Table 2. Information about endogenous target sites and associated potential off-target sites (provided as a separate file). Mismatched and bulged nucleotides in the off-target sites are indicated in red and blue lower-case fonts, respectively. Mismatched nucleotides at the 5' end of target sequences that are 20-nt away from the PAM were not counted as mismatches.

Supplementary Table 3. Training and test datasets used for the development of PEselector (provided as a separate file).

Supplementary Table 4. Oligonucleotides used in this study (provided as a separate file).

Supplementary Note

PE2 (addgene # 132775)

NLS
Cas9 H840A
Linker A
Reverse transcriptase

atgaaacggacagccgacggaagcgagttcgagtcaccaaagaagaagcggaaagtcgacaagaagtacagc
atcggcctggacatcggcaccaactctgtgggctgggccgtgatcaccgacgagtacaaggtgcccagcaagaaa
ttcaaggtgctgggcaacaccgaccggcacagcatcaagaagaacctgatcggagccctgctgttcgacagcggc
gaaacagccgaggccacccggctgaagagaaccgccagaagaagatacaccagacggaagaaccggatctg
ctatctgcaagagatcttcagcaacgagatggccaaggtggacgacagcttcttccacagactggaagagtccttcct
ggtggaagaggataagaagcacgagcggcaccccatcttcggcaacatcgtggacgaggtggcctaccacgaga
agtaccccaccatctaccacctgagaaagaaactggtggacagcaccgacaaggccgacctgcggctgatctatct
ggccctggcccacatgatcaagttccggggccacttcctgatcgagggcgacctgaaccccgacaacagcgacgt
ggacaagctgttcatccagctggtgcagacctacaaccagctgttcgaggaaaaccccatcaacgccagcggcgtg
gacgccaaggccatcctgtctgccagactgagcaagagcagacggctggaaaatctgatcgcccagctgcccggc
gagaagaagaatggcctgttcggaaacctgattgccctgagcctgggcctgacccccaacttcaagagcaacttcg
acctggccgaggatgccaaactgcagctgagcaaggacacctacgacgacgacctggacaacctgctggcccag
atcggcgaccagtacgccgacctgtttctggccgccaagaacctgtccgacgccatcctgctgagcgacatcctgag
agtgaacaccgagatcaccaaggcccccctgagcgcctctatgatcaagagatacgacgagcaccaccaggacc
tgaccctgctgaaagctctcgtgcggcagcagctgcctgagaagtacaaagagattttcttcgaccagagcaagaac
ggctacgccggctacattgacggcggagccagccaggaagagttctacaagttcatcaagcccatcctggaaaag
atggacggcaccgaggaactgctcgtgaagctgaacagagaggacctgctgcggaagcagcggaccttcgacaa
cggcagcatcccccaccagatccacctgggagagctgcacgccattctgcggcggcaggaagatttttacccattcc
tgaaggacaaccgggaaaagatcgagaagatcctgaccttccgcatcccctactacgtgggccctctggccaggg
gaaacagcagattcgcctggatgaccagaaagagcgaggaaaccatcaccccctggaacttcgaggaagtggtg
gacaagggcgcttccgcccagagcttcatcgagcggatgaccaacttcgataagaacctgcccaacgagaaggtg
ctgcccaagcacagcctgctgtacgagtacttcaccgtgtataacgagctgaccaaagtgaaatacgtgaccgagg
gaatgagaaagcccgccttcctgagcggcgagcagaaaaaggccatcgtggacctgctgttcaagaccaaccgg
aaagtgaccgtgaagcagctgaaagaggactacttcaagaaaatcgagtgcttcgactccgtggaaatctccggcg
tggaagatcggttcaacgcctccctgggcacataccacgatctgctgaaaattatcaaggacaaggacttcctggac
aatgaggaaaacgaggacattctggaagatatcgtgctgaccctgacactgtttgaggacagagagatgatcgagg
aacggctgaaaacctatgcccacctgttcgacgacaaagtgatgaagcagctgaagcggcggagatacaccggct
ggggcaggctgagccggaagctgatcaacggcatccgggacaagcagtccggcaagacaatcctggatttcctga
agtccgacggcttcgccaacagaaacttcatgcagctgatccacgacgacagcctgacctttaaagaggacatcca
gaaagcccaggtgtccggccagggcgatagcctgcacgagcacattgccaatctggccggcagccccgccattaa
gaagggcatcctgcagacagtgaaggtggtggacgagctcgtgaaagtgatgggccggcacaagcccgagaac
atcgtgatcgaaatggccagagagaaccagaccacccagaagggacagaagaacagccgcgagagaatgaa
gcggatcgaagagggcatcaaagagctgggcagccagatcctgaaagaacaccccgtggaaaacacccagctg
cagaacgagaagctgtacctgtactacctgcagaatgggcgggatatgtacgtggaccaggaactggacatcaac
cggctgtccgactacgatgtggacgctatcgtgcctcagagctttctgaaggacgactccatcgacaacaaggtgctg
accagaagcgacaagaaccggggcaagagcgacaacgtgccctccgaagaggtcgtgaagaagatgaagaa
ctactggcggcagctgctgaacgccaagctgattacccagagaaagttcgacaatctgaccaaggccgagagagg
cggcctgagcgaactggataaggccggcttcatcaagagacagctggtggaaacccggcagatcacaaagcacg

tggcacagatcctggactcccggatgaacactaagtacgacgagaatgacaagctgatccgggaagtgaaagtga
tcaccctgaagtccaagctggtgtccgatttccggaaggatttccagttttacaaagtgcgcgagatcaacaactacca
ccacgcccacgacgcctacctgaacgccgtcgtgggaaccgccctgatcaaaaagtaccctaagctggaaagcg
agttcgtgtacggcgactacaaggtgtacgacgtgcggaagatgatcgccaagagcgagcaggaaatcggcaag
gctaccgccaagtacttcttctacagcaacatcatgaactttttcaagaccgagattaccctggccaacggcgagatcc
ggaagcggcctctgatcgagacaaacggcgaaaccggggagatcgtgtgggataagggccgggattttgccacc
gtgcggaaagtgctgagcatgccccaagtgaatatcgtgaaaaagaccgaggtgcagacaggcggcttcagcaa
agagtctatcctgcccaagaggaacagcgataagctgatcgccagaaagaaggactgggaccctaagaagtacg
gcggcttcgacagccccaccgtggcctattctgtgctggtggtggccaaagtggaaaagggcaagtccaagaaact
gaagagtgtgaaagagctgctggggatcaccatcatggaaagaagcagcttcgagaagaatcccatcgactttctg
gaagccaagggctacaaagaagtgaaaaaggacctgatcatcaagctgcctaagtactccctgttcgagctggaa
aacggccggaagagaatgctggcctctgccggcgaactgcagaagggaaacgaactggccctgccctccaaata
tgtgaacttcctgtacctggccagccactatgagaagctgaagggctcccccgaggataatgagcagaaacagctgt
ttgtggaacagcacaagcactacctggacgagatcatcgagcagatcagcgagttctccaagagagtgatcctggc
cgacgctaatctggacaaagtgctgtccgcctacaacaagcaccgggataagcccatcagagagcaggccgaga
atatcatccacctgtttaccctgaccaatctgggagcccctgccgccttcaagtactttgacaccaccatcgaccggaa
gaggtacaccagcaccaaagaggtgctggacgccaccctgatccaccagagcatcaccggcctgtacgagacac
ggatcgacctgtctcagctgggaggtgactctggaggatctagcggaggatcctctggcagcgagacaccaggaac
aagcgagtcagcaacaccagagagcagtggcggcagcagcggcggcagcagcaccctaaatatagaagatga
gtatcggctacatgagacctcaaaagagccagatgtttctctagggtccacatggctgtctgattttcctcaggcctggg
cggaaaccgggggcatgggactggcagttcgccaagctcctctgatcatacctctgaaagcaacctctacccccgtg
tccataaaacaataccccatgtcacaagaagccagactggggatcaagccccacatacagagactgttggaccag
ggaatactggtaccctgccagtcccccctggaacacgcccctgctacccgttaagaaaccagggactaatgattatag
gcctgtccaggatctgagagaagtcaacaagcgggtggaagacatccaccccaccgtgcccaacccttacaacct
cttgagcgggctcccaccgtcccaccagtggtacactgtgcttgatttaaaggatgcctttttctgcctgagactccaccc
caccagtcagcctctcttcgcctttgagtggagagatccagagatgggaatctcaggacaattgacctggaccagact
cccacagggtttcaaaaacagtcccaccctgtttaatgaggcactgcacagagacctagcagacttccggatccagc
acccagacttgatcctgctacagtacgtggatgacttactgctggccgccacttctgagctagactgccaacaaggtac
tcgggccctgttacaaaccctagggaacctcgggtatcgggcctcggccaagaaagcccaaatttgccagaaaca
ggtcaagtatctggggtatcttctaaaagagggtcagagatggctgactgaggccagaaaagagactgtgatgggg
cagcctactccgaagacccctcgacaactaagggagttcctagggaaggcaggcttctgtcgcctcttcatccctggg
tttgcagaaatggcagcccccctgtaccctctcaccaaaccggggactctgtttaattggggcccagaccaacaaaa
ggcctatcaagaaatcaagcaagctcttctaactgccccagccctggggttgccagatttgactaagccctttgaactct
ttgtcgacgagaagcagggctacgccaaaggtgtcctaacgcaaaaactgggaccttggcgtcggccggtggccta
cctgtccaaaaagctagacccagtagcagctgggtggcccccttgcctacggatggtagcagccattgccgtactga
caaaggatgcaggcaagctaaccatgggacagccactagtcattctggcccccccatgcagtagaggcactagtca
aacaaccccccgaccgctggctttccaacgcccggatgactcactatcaggccttgcttttggacacggaccgggtcc
agttcggaccggtggtagccctgaacccggctacgctgctcccactgcctgaggaagggctgcaacacaactgcctt
gatatcctggccgaagcccacggaacccgacccgacctaacggaccagccgctcccagacgccgaccacacct
ggtacacggatggaagcagtctcttacaagagggacagcgtaaggcgggagctgcggtgaccaccgagaccga
ggtaatctgggctaaagccctgccagccgggacatccgctcagcgggctgaactgatagcactcacccaggcccta
aagatggcagaaggtaagaagctaaatgtttatactgatagccgttatgcttttgctactgcccatatccatggagaaat
atacagaaggcgtgggtggctcacatcagaaggcaaagagatcaaaaataaagacgagatcttggccctactaaa
agccctctttctgcccaaaagacttagcataatccattgtccaggacatcaaaagggacacagcgccgaggctaga
ggcaaccggatggctgaccaagcggcccgaaaggcagccatcacagagactccagacacctctaccctcctcat
agaaaattcatcaccctctggcggctcaaaaagaaccgccgacggcagcgaattcgagcccaagaagaagagg

aaagtctaa

PE2-mid_RPA70

NLS
Cas9 H840A
Linker A
Linker B
RPA70 ssDBD
Reverse transcriptase

atgaaacggacagccgacggaagcgagttcgagtcaccaaagaagaagcggaaagtcgacaagaagtacagc
atcggcctggacatcggcaccaactctgtgggctgggccgtgatcaccgacgagtacaaggtgcccagcaagaaa
ttcaaggtgctgggcaacaccgaccggcacagcatcaagaagaacctgatcggagccctgctgttcgacagcggc
gaaacagccgaggccacccggctgaagagaaccgccagaagaagatacaccagacggaagaaccggatctg
ctatctgcaagagatcttcagcaacgagatggccaaggtggacgacagcttcttccacagactggaagagtccttcct
ggtggaagaggataagaagcacgagcggcaccccatcttcggcaacatcgtggacgaggtggcctaccacgaga
agtaccccaccatctaccacctgagaaagaaactggtggacagcaccgacaaggccgacctgcggctgatctatct
ggccctggcccacatgatcaagttccggggccacttcctgatcgagggcgacctgaaccccgacaacagcgacgt
ggacaagctgttcatccagctggtgcagacctacaaccagctgttcgaggaaaaccccatcaacgccagcggcgtg
gacgccaaggccatcctgtctgccagactgagcaagagcagacggctggaaaatctgatcgcccagctgcccggc
gagaagaagaatggcctgttcggaaacctgattgccctgagcctgggcctgacccccaacttcaagagcaacttcg
acctggccgaggatgccaaactgcagctgagcaaggacacctacgacgacgacctggacaacctgctggcccag
atcggcgaccagtacgccgacctgtttctggccgccaagaacctgtccgacgccatcctgctgagcgacatcctgag
agtgaacaccgagatcaccaaggcccccctgagcgcctctatgatcaagagatacgacgagcaccaccaggacc
tgaccctgctgaaagctctcgtgcggcagcagctgcctgagaagtacaaagagattttcttcgaccagagcaagaac
ggctacgccggctacattgacggcggagccagccaggaagagttctacaagttcatcaagcccatcctggaaaag
atggacggcaccgaggaactgctcgtgaagctgaacagagaggacctgctgcggaagcagcggaccttcgacaa
cggcagcatcccccaccagatccacctgggagagctgcacgccattctgcggcggcaggaagatttttacccattcc
tgaaggacaaccgggaaaagatcgagaagatcctgaccttccgcatcccctactacgtgggccctctggccaggg
gaaacagcagattcgcctggatgaccagaaagagcgaggaaaccatcaccccctggaacttcgaggaagtggtg
gacaagggcgcttccgcccagagcttcatcgagcggatgaccaacttcgataagaacctgcccaacgagaaggtg
ctgcccaagcacagcctgctgtacgagtacttcaccgtgtataacgagctgaccaaagtgaaatacgtgaccgagg
gaatgagaaagcccgccttcctgagcggcgagcagaaaaaggccatcgtggacctgctgttcaagaccaaccgg
aaagtgaccgtgaagcagctgaaagaggactacttcaagaaaatcgagtgcttcgactccgtggaaatctccggcg
tggaagatcggttcaacgcctccctgggcacataccacgatctgctgaaaattatcaaggacaaggacttcctggac
aatgaggaaaacgaggacattctggaagatatcgtgctgaccctgacactgtttgaggacagagagatgatcgagg
aacggctgaaaacctatgcccacctgttcgacgacaaagtgatgaagcagctgaagcggcggagatacaccggct
ggggcaggctgagccggaagctgatcaacggcatccgggacaagcagtccggcaagacaatcctggatttcctga
agtccgacggcttcgccaacagaaacttcatgcagctgatccacgacgacagcctgacctttaaagaggacatcca
gaaagcccaggtgtccggccagggcgatagcctgcacgagcacattgccaatctggccggcagccccgccattaa
gaagggcatcctgcagacagtgaaggtggtggacgagctcgtgaaagtgatgggccggcacaagcccgagaac
atcgtgatcgaaatggccagagagaaccagaccacccagaagggacagaagaacagccgcgagagaatgaa
gcggatcgaagagggcatcaaagagctgggcagccagatcctgaaagaacaccccgtggaaaacacccagctg
cagaacgagaagctgtacctgtactacctgcagaatgggcgggatatgtacgtggaccaggaactggacatcaac
cggctgtccgactacgatgtggacgctatcgtgcctcagagctttctgaaggacgactccatcgacaacaaggtgctg
accagaagcgacaagaaccggggcaagagcgacaacgtgccctccgaagaggtcgtgaagaagatgaagaa

ctactggcggcagctgctgaacgccaagctgattacccagagaaagttcgacaatctgaccaaggccgagagagg
cggcctgagcgaactggataaggccggcttcatcaagagacagctggtggaaacccggcagatcacaaagcacg
tggcacagatcctggactcccggatgaacactaagtacgacgagaatgacaagctgatccgggaagtgaaagtga
tcaccctgaagtccaagctggtgtccgatttccggaaggatttccagttttacaaagtgcgcgagatcaacaactacca
ccacgcccacgacgcctacctgaacgccgtcgtgggaaccgccctgatcaaaaagtaccctaagctggaaagcg
agttcgtgtacggcgactacaaggtgtacgacgtgcggaagatgatcgccaagagcgagcaggaaatcggcaag
gctaccgccaagtacttcttctacagcaacatcatgaactttttcaagaccgagattaccctggccaacggcgagatcc
ggaagcggcctctgatcgagacaaacggcgaaaccggggagatcgtgtgggataagggccgggattttgccacc
gtgcggaaagtgctgagcatgccccaagtgaatatcgtgaaaaagaccgaggtgcagacaggcggcttcagcaa
agagtctatcctgcccaagaggaacagcgataagctgatcgccagaaagaaggactgggaccctaagaagtacg
gcggcttcgacagccccaccgtggcctattctgtgctggtggtggccaaagtggaaaagggcaagtccaagaaact
gaagagtgtgaaagagctgctggggatcaccatcatggaaagaagcagcttcgagaagaatcccatcgactttctg
gaagccaagggctacaaagaagtgaaaaaggacctgatcatcaagctgcctaagtactccctgttcgagctggaa
aacggccggaagagaatgctggcctctgccggcgaactgcagaagggaaacgaactggccctgccctccaaata
tgtgaacttcctgtacctggccagccactatgagaagctgaagggctcccccgaggataatgagcagaaacagctgt
ttgtggaacagcacaagcactacctggacgagatcatcgagcagatcagcgagttctccaagagagtgatcctggc
cgacgctaatctggacaaagtgctgtccgcctacaacaagcaccgggataagcccatcagagagcaggccgaga
atatcatccacctgtttaccctgaccaatctgggagcccctgccgccttcaagtactttgacaccaccatcgaccggaa
gaggtacaccagcaccaaagaggtgctggacgccaccctgatccaccagagcatcaccggcctgtacgagacac
ggatcgacctgtctcagctgggaggtgacTCTGGTGGTTCTCCCAAGAAGAAGAGGAAAGTC
GGTAGTTCCGGATCTggagggagtaacaccaactggaaaaccttgtatgaggtcaaatccgagaacctg
ggccaaggcgacaagccggactactttagttctgtggccacagtggtgtatcttcgcaaagagaactgcatgtaccaa
gcctgcccgactcaggactgcaataagaaagtgattgatcaacagaatggattgtaccgctgtgagaagtgcgaca
ccgaatttcccaatttcaagtaccgcatgatcctgtcagtaaatattgcagattttcaagagaatcagtgggtgacttgttt
ccaggagtctgctgaagctatccttggacaaaatgctgcttatcttggggaattaaaagacaagaatgaacaggcattt
gaagaagttttccagaatgccaacttccgatctttcatattcagagtcagggtcaaagtggagacctacaacgacgag
tctcgaattaaggccactgtgatggacgtgaagcccgtggactacagagagtatggccgaaggctggtcatgagcat
caggagaagtgcattgatgtctggaggatctagcggaggatcctctggcagcgagacaccaggaacaagcgagtc
agcaacaccagagagcagtggcggcagcagcggcggcagcagcaccctaaatatagaagatgagtatcggcta
catgagacctcaaaagagccagatgtttctctagggtccacatggctgtctgattttcctcaggcctgggcggaaaccg
gggcatgggactggcagttcgccaagctcctctgatcatacctctgaaagcaacctctacccccgtgtccataaaac
aataccccatgtcacaagaagccagactggggatcaagccccacatacagagactgttggaccagggaatactgg
taccctgccagtcccccctggaacacgcccctgctacccgttaagaaaccagggactaatgattataggcctgtccag
gatctgagagaagtcaacaagcgggtggaagacatccaccccaccgtgcccaacccttacaacctcttgagcggg
ctcccaccgtcccaccagtggtacactgtgcttgatttaaaggatgcctttttctgcctgagactccaccccaccagtcag
cctctcttcgcctttgagtggagagatccagagatgggaatctcaggacaattgacctggaccagactcccacagggt
ttcaaaaacagtcccaccctgtttaatgaggcactgcacagagacctagcagacttccggatccagcacccagactt
gatcctgctacagtacgtggatgacttactgctggccgccacttctgagctagactgccaacaaggtactcgggccctg
ttacaaaccctagggaacctcgggtatcgggcctcggccaagaaagcccaaatttgccagaaacaggtcaagtatc
tggggtatcttctaaaagagggtcagagatggctgactgaggccagaaaagagactgtgatggggcagcctactcc
gaagacccctcgacaactaagggagttcctagggaaggcaggcttctgtcgcctcttcatccctgggtttgcagaaat
ggcagcccccctgtaccctctcaccaaaccggggactctgtttaattggggcccagaccaacaaaaggcctatcaa
gaaatcaagcaagctcttctaactgccccagccctggggttgccagatttgactaagccctttgaactctttgtcgacga
gaagcagggctacgccaaaggtgtcctaacgcaaaaactgggaccttggcgtcggccggtggcctacctgtccaa
aaagctagacccagtagcagctgggtggcccccttgcctacggatggtagcagccattgccgtactgacaaaggat
gcaggcaagctaaccatgggacagccactagtcattctggccccccatgcagtagaggcactagtcaaacaaccc

cccgaccgctggctttccaacgcccggatgactcactatcaggccttgcttttggacacggaccgggtccagttcgga
ccggtggtagccctgaacccggctacgctgctcccactgcctgaggaagggctgcaacacaactgccttgatatcct
ggccgaagcccacggaacccgacccgacctaacggaccagccgctcccagacgccgaccacacctggtacac
ggatggaagcagtctcttacaagagggacagcgtaaggcgggagctgcggtgaccaccgagaccgaggtaatct
gggctaaagccctgccagccgggacatccgctcagcgggctgaactgatagcactcacccaggccctaaagatgg
cagaaggtaagaagctaaatgtttatactgatagccgttatgcttttgctactgcccatatccatggagaaatatacaga
aggcgtgggtggctcacatcagaaggcaaagagatcaaaaataaagacgagatcttggccctactaaaagccctc
tttctgcccaaaagacttagcataatccattgtccaggacatcaaaagggacacagcgccgaggctagaggcaacc
ggatggctgaccaagcggcccgaaaggcagccatcacagagactccagacacctctaccctcctcatagaaaatt
catcaccctctggcggctcaaaaagaaccgccgacggcagcgaattcgagcccaagaagaagaggaaagtcta
a

hyPE2 = PE2-mid_Rad51

NLS
Cas9 H840A
Linker A
Linker B
Rad51 ssDBD
Reverse transcriptase

atgaaacggacagccgacggaagcgagttcgagtcaccaaagaagaagcggaaagtcgacaagaagtacagc
atcggcctggacatcggcaccaactctgtgggctgggccgtgatcaccgacgagtacaaggtgcccagcaagaaa
ttcaaggtgctgggcaacaccgaccggcacagcatcaagaagaacctgatcggagccctgctgttcgacagcggc
gaaacagccgaggccacccggctgaagagaaccgccagaagaagatacaccagacggaagaaccggatctg
ctatctgcaagagatcttcagcaacgagatggccaaggtggacgacagcttcttccacagactggaagagtccttcct
ggtggaagaggataagaagcacgagcggcaccccatcttcggcaacatcgtggacgaggtggcctaccacgaga
agtaccccaccatctaccacctgagaaagaaactggtggacagcaccgacaaggccgacctgcggctgatctatct
ggccctggcccacatgatcaagttccggggccacttcctgatcgagggcgacctgaaccccgacaacagcgacgt
ggacaagctgttcatccagctggtgcagacctacaaccagctgttcgaggaaaaccccatcaacgccagcggcgtg
gacgccaaggccatcctgtctgccagactgagcaagagcagacggctggaaaatctgatcgcccagctgcccggc
gagaagaagaatggcctgttcggaaacctgattgccctgagcctgggcctgacccccaacttcaagagcaacttcg
acctggccgaggatgccaaactgcagctgagcaaggacacctacgacgacgacctggacaacctgctggcccag
atcggcgaccagtacgccgacctgtttctggccgccaagaacctgtccgacgccatcctgctgagcgacatcctgag
agtgaacaccgagatcaccaaggcccccctgagcgcctctatgatcaagagatacgacgagcaccaccaggacc
tgaccctgctgaaagctctcgtgcggcagcagctgcctgagaagtacaaagagattttcttcgaccagagcaagaac
ggctacgccggctacattgacggcggagccagccaggaagagttctacaagttcatcaagcccatcctggaaaag
atggacggcaccgaggaactgctcgtgaagctgaacagagaggacctgctgcggaagcagcggaccttcgacaa
cggcagcatcccccaccagatccacctgggagagctgcacgccattctgcggcggcaggaagatttttacccattcc
tgaaggacaaccgggaaaagatcgagaagatcctgaccttccgcatcccctactacgtgggccctctggccaggg
gaaacagcagattcgcctggatgaccagaaagagcgaggaaaccatcaccccctggaacttcgaggaagtggtg
gacaagggcgcttccgcccagagcttcatcgagcggatgaccaacttcgataagaacctgcccaacgagaaggtg
ctgcccaagcacagcctgctgtacgagtacttcaccgtgtataacgagctgaccaaagtgaaatacgtgaccgagg
gaatgagaaagcccgccttcctgagcggcgagcagaaaaaggccatcgtggacctgctgttcaagaccaaccgg
aaagtgaccgtgaagcagctgaaagaggactacttcaagaaaatcgagtgcttcgactccgtggaaatctccggcg

tggaagatcggttcaacgcctccctgggcacataccacgatctgctgaaaattatcaaggacaaggacttcctggac
aatgaggaaaacgaggacattctggaagatatcgtgctgaccctgacactgtttgaggacagagagatgatcgagg
aacggctgaaaacctatgcccacctgttcgacgacaaagtgatgaagcagctgaagcggcggagatacaccggct
ggggcaggctgagccggaagctgatcaacggcatccgggacaagcagtccggcaagacaatcctggatttcctga
agtccgacggcttcgccaacagaaacttcatgcagctgatccacgacgacagcctgacctttaaagaggacatcca
gaaagcccaggtgtccggccagggcgatagcctgcacgagcacattgccaatctggccggcagccccgccattaa
gaagggcatcctgcagacagtgaaggtggtggacgagctcgtgaaagtgatgggccggcacaagcccgagaac
atcgtgatcgaaatggccagagagaaccagaccacccagaagggacagaagaacagccgcgagagaatgaa
gcggatcgaagagggcatcaaagagctgggcagccagatcctgaaagaacaccccgtggaaaacacccagctg
cagaacgagaagctgtacctgtactacctgcagaatgggcgggatatgtacgtggaccaggaactggacatcaac
cggctgtccgactacgatgtggacgctatcgtgcctcagagctttctgaaggacgactccatcgacaacaaggtgctg
accagaagcgacaagaaccggggcaagagcgacaacgtgccctccgaagaggtcgtgaagaagatgaagaa
ctactggcggcagctgctgaacgccaagctgattacccagagaaagttcgacaatctgaccaaggccgagagagg
cggcctgagcgaactggataaggccggcttcatcaagagacagctggtggaaacccggcagatcacaaagcacg
tggcacagatcctggactcccggatgaacactaagtacgacgagaatgacaagctgatccgggaagtgaaagtga
tcaccctgaagtccaagctggtgtccgatttccggaaggatttccagttttacaaagtgcgcgagatcaacaactacca
ccacgcccacgacgcctacctgaacgccgtcgtgggaaccgccctgatcaaaaagtaccctaagctggaaagcg
agttcgtgtacggcgactacaaggtgtacgacgtgcggaagatgatcgccaagagcgagcaggaaatcggcaag
gctaccgccaagtacttcttctacagcaacatcatgaactttttcaagaccgagattaccctggccaacggcgagatcc
ggaagcggcctctgatcgagacaaacggcgaaaccggggagatcgtgtgggataagggccgggattttgccacc
gtgcggaaagtgctgagcatgccccaagtgaatatcgtgaaaaagaccgaggtgcagacaggcggcttcagcaa
agagtctatcctgcccaagaggaacagcgataagctgatcgccagaaagaaggactgggaccctaagaagtacg
gcggcttcgacagccccaccgtggcctattctgtgctggtggtggccaaagtggaaaagggcaagtccaagaaact
gaagagtgtgaaagagctgctggggatcaccatcatggaaagaagcagcttcgagaagaatcccatcgactttctg
gaagccaagggctacaaagaagtgaaaaaggacctgatcatcaagctgcctaagtactccctgttcgagctggaa
aacggccggaagagaatgctggcctctgccggcgaactgcagaagggaaacgaactggccctgccctccaaata
tgtgaacttcctgtacctggccagccactatgagaagctgaagggctcccccgaggataatgagcagaaacagctgt
ttgtggaacagcacaagcactacctggacgagatcatcgagcagatcagcgagttctccaagagagtgatcctggc
cgacgctaatctggacaaagtgctgtccgcctacaacaagcaccgggataagcccatcagagagcaggccgaga
atatcatccacctgtttaccctgaccaatctgggagcccctgccgccttcaagtactttgacaccaccatcgaccggaa
gaggtacaccagcaccaaagaggtgctggacgccaccctgatccaccagagcatcaccggcctgtacgagacac
ggatcgacctgtctcagctgggaggtgacTCTGGTGGTTCTCCCAAGAAGAAGAGGAAAGTC
GGTAGTTCCGGATCTgcaatgcagatgcagcttgaagcaaatgcagatacttcagtggaagaagaaagct
ttggcccacaacccatttcacggttagagcagtgtggcataaatgccaacgatgtgaagaaattggaagaagctgga
ttccatactgtggaggctgttgcctatgcgccaaagaaggagctaataaatattaagggaattagtgaagccaaagct
gataaaattctggctgaggcagctaaattagttccaatgggtttcaccactgcaactgaattccaccaaaggcggtca
gagatcatacagattactactggctccaaagagcttgacaaactacttcaatctggaggatctagcggaggatcctct
ggcagcgagacaccaggaacaagcgagtcagcaacaccagagagcagtggcggcagcagcggcggcagca
gcaccctaaatatagaagatgagtatcggctacatgagacctcaaaagagccagatgtttctctagggtccacatgg
ctgtctgattttcctcaggcctgggcggaaaccgggggcatgggactggcagttcgccaagctcctctgatcatacctct
gaaagcaacctctacccccgtgtccataaaacaataccccatgtcacaagaagccagactggggatcaagcccca
catacagagactgttggaccagggaatactggtaccctgccagtccccctggaacacgcccctgctacccgttaaga
aaccagggactaatgattataggcctgtccaggatctgagagaagtcaacaagcgggtggaagacatccacccca
ccgtgcccaacccttacaacctcttgagcgggctcccaccgtcccaccagtggtacactgtgcttgatttaaaggatgc
ctttttctgcctgagactccaccccaccagtcagcctctcttcgcctttgagtggagagatccagagatgggaatctcag
gacaattgacctggaccagactcccacagggtttcaaaaacagtcccaccctgtttaatgaggcactgcacagaga

cctagcagacttccggatccagcacccagacttgatcctgctacagtacgtggatgacttactgctggccgccacttct
gagctagactgccaacaaggtactcgggccctgttacaaaccctagggaacctcgggtatcgggcctcggccaag
aaagcccaaatttgccagaaacaggtcaagtatctggggtatcttctaaaagagggtcagagatggctgactgaggc
cagaaaagagactgtgatggggcagcctactccgaagacccctcgacaactaagggagttcctagggaaggcag
gcttctgtcgcctcttcatccctgggtttgcagaaatggcagcccccctgtaccctctcaccaaaccggggactctgttta
attggggcccagaccaacaaaaggcctatcaagaaatcaagcaagctcttctaactgccccagccctggggttgcc
agatttgactaagccctttgaactctttgtcgacgagaagcagggctacgccaaaggtgtcctaacgcaaaaactgg
gaccttggcgtcggccggtggcctacctgtccaaaaagctagacccagtagcagctgggtggcccccttgcctacgg
atggtagcagccattgccgtactgacaaaggatgcaggcaagctaaccatgggacagccactagtcattctggccc
cccatgcagtagaggcactagtcaaacaacccccgaccgctggctttccaacgcccggatgactcactatcaggc
cttgcttttggacacggaccgggtccagttcggaccggtggtagccctgaacccggctacgctgctcccactgcctga
ggaagggctgcaacacaactgccttgatatcctggccgaagcccacggaacccgacccgacctaacggaccagc
cgctcccagacgccgaccacacctggtacacggatggaagcagtctcttacaagagggacagcgtaaggcggga
gctgcggtgaccaccgagaccgaggtaatctgggctaaagccctgccagccgggacatccgctcagcgggctgaa
ctgatagcactcacccaggccctaaagatggcagaaggtaagaagctaaatgtttatactgatagccgttatgcttttg
ctactgcccatatccatggagaaatatacagaaggcgtgggtggctcacatcagaaggcaaagagatcaaaaata
aagacgagatcttggccctactaaaagccctctttctgcccaaaagacttagcataatccattgtccaggacatcaaa
agggacacagcgccgaggctagaggcaaccggatggctgaccaagcggcccgaaaggcagccatcacagag
actccagacacctctaccctcctcatagaaaattcatcaccctctggcggctcaaaaagaaccgccgacggcagcg
aattcgagcccaagaagaagaggaaagtctaa

PE2-N_Rad51

NLS
Cas9 H840A
Linker A
Linker B
Rad51 ssDBD
Reverse transcriptase

atgaaacggacagccgacggaagcgagttcgagtcaccaaagaagaagcggaaagtcgcaatgcagatgcag
cttgaagcaaatgcagatacttcagtggaagaagaaagctttggcccacaacccatttcacggttagagcagtgtgg
cataaatgccaacgatgtgaagaaattggaagaagctggattccatactgtggaggctgttgcctatgcgccaaaga
aggagctaataaatattaagggaattagtgaagccaaagctgataaaattctggctgaggcagctaaattagttccaa
tgggtttcaccactgcaactgaattccaccaaaggcggtcagagatcatacagattactactggctccaaagagcttg
acaaactacttcaaTCTGGTGGTTCTCCCAAGAAGAAGAGGAAAGTCGGTAGTTCCGG
ATCTgacaagaagtacagcatcggcctggacatcggcaccaactctgtgggctgggccgtgatcaccgacgagt
acaaggtgcccagcaagaaattcaaggtgctgggcaacaccgaccggcacagcatcaagaagaacctgatcgg
agccctgctgttcgacagcggcgaaacagccgaggccacccggctgaagagaaccgccagaagaagatacacc
agacggaagaaccggatctgctatctgcaagagatcttcagcaacgagatggccaaggtggacgacagcttcttcc
acagactggaagagtccttcctggtggaagaggataagaagcacgagcggcaccccatcttcggcaacatcgtgg
acgaggtggcctaccacgagaagtaccccaccatctaccacctgagaaagaaactggtggacagcaccgacaa
ggccgacctgcggctgatctatctggccctggcccacatgatcaagttccggggccacttcctgatcgagggcgacct
gaaccccgacaacagcgacgtggacaagctgttcatccagctggtgcagacctacaaccagctgttcgaggaaaa
ccccatcaacgccagcggcgtggacgccaaggccatcctgtctgccagactgagcaagagcagacggctggaaa

atctgatcgcccagctgcccggcgagaagaagaatggcctgttcggaaacctgattgccctgagcctgggcctgacc
cccaacttcaagagcaacttcgacctggccgaggatgccaaactgcagctgagcaaggacacctacgacgacga
cctggacaacctgctggcccagatcggcgaccagtacgccgacctgtttctggccgccaagaacctgtccgacgcc
atcctgctgagcgacatcctgagagtgaacaccgagatcaccaaggccccccctgagcgcctctatgatcaagagat
acgacgagcaccaccaggacctgaccctgctgaaagctctcgtgcggcagcagctgcctgagaagtacaaagag
attttcttcgaccagagcaagaacggctacgccggctacattgacggcggagccagccaggaagagttctacaagtt
catcaagcccatcctggaaaagatggacggcaccgaggaactgctcgtgaagctgaacagagaggacctgctgc
ggaagcagcggaccttcgacaacggcagcatcccccaccagatccacctgggagagctgcacgccattctgcgg
cggcaggaagatttttacccattcctgaaggacaaccgggaaaagatcgagaagatcctgaccttccgcatcccct a
ctacgtgggccctctggccaggggaaacagcagattcgcctggatgaccagaaagagcgaggaaaccatcaccc
cctggaacttcgaggaagtggtggacaagggcgcttccgcccagagcttcatcgagcggatgaccaacttcgataa
gaacctgcccaacgagaaggtgctgcccaagcacagcctgctgtacgagtacttcaccgtgtataacgagctgacc
aaagtgaaatacgtgaccgagggaatgagaaagcccgccttcctgagcggcgagcagaaaaaggccatcgtgg
acctgctgttcaagaccaaccggaaagtgaccgtgaagcagctgaaagaggactacttcaagaaaatcgagtgctt
cgactccgtggaaatctccggcgtggaagatcggttcaacgcctccctgggcacataccacgatctgctgaaaattat
caaggacaaggacttcctggacaatgaggaaaacgaggacattctggaagatatcgtgctgaccctgacactgtttg
aggacagagagatgatcgaggaacggctgaaaacctatgcccacctgttcgacgacaaagtgatgaagcagctg
aagcggcggagatacaccggctggggcaggctgagccggaagctgatcaacggcatccgggacaagcagtccg
gcaagacaatcctggatttcctgaagtccgacggcttcgccaacagaaacttcatgcagctgatccacgacgacagc
ctgacctttaaagaggacatccagaaagcccaggtgtccggccagggcgatagcctgcacgagcacattgccaat
ctggccggcagccccgccattaagaagggcatcctgcagacagtgaaggtggtggacgagctcgtgaaagtgatg
ggccggcacaagcccgagaacatcgtgatcgaaatggccagagagaaccagaccacccagaagggacagaa
gaacagccgcgagagaatgaagcggatcgaagagggcatcaaagagctgggcagccagatcctgaaagaaca
ccccgtggaaaacacccagctgcagaacgagaagctgtacctgtactacctgcagaatgggcgggatatgtacgtg
gaccaggaactggacatcaaccggctgtccgactacgatgtggacgctatcgtgcctcagagctttctgaaggacga
ctccatcgacaacaaggtgctgaccagaagcgacaagaaccggggcaagagcgacaacgtgccctccgaaga
ggtcgtgaagaagatgaagaactactggcggcagctgctgaacgccaagctgattacccagagaaagttcgacaa
tctgaccaaggccgagagaggcggcctgagcgaactggataaggccggcttcatcaagagacagctggtggaaa
cccggcagatcacaaagcacgtggcacagatcctggactcccggatgaacactaagtacgacgagaatgacaag
ctgatccgggaagtgaaagtgatcaccctgaagtccaagctggtgtccgatttccggaaggatttccagttttacaaag
tgcgcgagatcaacaactaccaccacgcccacgacgcctacctgaacgccgtcgtgggaaccgccctgatcaaaa
agtaccctaagctggaaagcgagttcgtgtacggcgactacaaggtgtacgacgtgcggaagatgatcgccaaga
gcgagcaggaaatcggcaaggctaccgccaagtacttcttctacagcaacatcatgaactttttcaagaccgagatta
ccctggccaacggcgagatccggaagcggcctctgatcgagacaaacggcgaaaccggggagatcgtgtgggat
aagggccgggattttgccaccgtgcggaaagtgctgagcatgccccaagtgaatatcgtgaaaaagaccgaggtg
cagacaggcggcttcagcaaagagtctatcctgcccaagaggaacagcgataagctgatcgccagaaagaagg
actgggaccctaagaagtacggcggcttcgacagccccaccgtggcctattctgtgctggtggtggccaaagtggaa
aagggcaagtccaagaaactgaagagtgtgaaagagctgctggggatcaccatcatggaaagaagcagcttcga
gaagaatcccatcgactttctggaagccaagggctacaaagaagtgaaaaaggacctgatcatcaagctgcctaa
gtactccctgttcgagctggaaaacggccggaagagaatgctggcctctgccggcgaactgcagaagggaaacga
actggccctgccctccaaatatgtgaacttcctgtacctggccagccactatgagaagctgaagggctcccccgagg
ataatgagcagaaacagctgtttgtggaacagcacaagcactacctggacgagatcatcgagcagatcagcgagtt
ctccaagagagtgatcctggccgacgctaatctggacaaagtgctgtccgcctacaacaagcaccgggataagccc
atcagagagcaggccgagaatatcatccacctgtttaccctgaccaatctgggagcccctgccgccttcaagtactttg
acaccaccatcgaccggaagaggtacaccagcaccaaagaggtgctggacgccaccctgatccaccagagcat
caccggcctgtacgagacacggatcgacctgtctcagctgggaggtgactctggaggatctagcggaggatcctctg

gcagcgagacaccaggaacaagcgagtcagcaacaccagagagcagtggcggcagcagcggcggcagcag
caccctaaatatagaagatgagtatcggctacatgagacctcaaaagagccagatgtttctctagggtccacatggct
gtctgattttcctcaggcctgggcggaaaccgggggcatgggactggcagttcgccaagctcctctgatcatacctctg
aaagcaacctctaccccgtgtccataaaacaataccccatgtcacaagaagccagactggggatcaagccccac
atacagagactgttggaccagggaatactggtaccctgccagtcccctggaacacgcccctgctacccgttaagaa
accagggactaatgattataggcctgtccaggatctgagagaagtcaacaagcgggtggaagacatccaccccac
cgtgcccaacccttacaacctcttgagcgggctcccaccgtcccaccagtggtacactgtgcttgatttaaaggatgcc
tttttctgcctgagactccaccccaccagtcagcctctcttcgcctttgagtggagagatccagagatgggaatctcagg
acaattgacctggaccagactcccacagggtttcaaaaacagtcccaccctgtttaatgaggcactgcacagagacc
tagcagacttccggatccagcacccagacttgatcctgctacagtacgtggatgacttactgctggccgccacttctga
gctagactgccaacaaggtactcgggccctgttacaaaccctagggaacctcgggtatcgggcctcggccaagaa
agcccaaatttgccagaaacaggtcaagtatctggggtatcttctaaaagagggtcagagatggctgactgaggcca
gaaaagagactgtgatggggcagcctactccgaagacccctcgacaactaagggagttcctagggaaggcaggc
ttctgtcgcctcttcatccctgggtttgcagaaatggcagcccccctgtaccctctcaccaaaccggggactctgtttaatt
ggggcccagaccaacaaaaggcctatcaagaaatcaagcaagctcttctaactgccccagccctggggttgccag
atttgactaagccctttgaactctttgtcgacgagaagcagggctacgccaaaggtgtcctaacgcaaaaactgggac
cttggcgtcggccggtggcctacctgtccaaaaagctagacccagtagcagctgggtggcccccttgcctacggatg
gtagcagccattgccgtactgacaaaggatgcaggcaagctaaccatgggacagccactagtcattctggcccccc
atgcagtagaggcactagtcaaacaacccccgaccgctggctttccaacgcccggatgactcactatcaggccttg
cttttggacacggaccgggtccagttcggaccggtggtagccctgaacccggctacgctgctcccactgcctgagga
agggctgcaacacaactgccttgatatcctggccgaagcccacggaacccgacccgacctaacggaccagccgct
cccagacgccgaccacacctggtacacggatggaagcagtctcttacaagagggacagcgtaaggcgggagctg
cggtgaccaccgagaccgaggtaatctgggctaaagccctgccagccgggacatccgctcagcgggctgaactga
tagcactcacccaggccctaaagatggcagaaggtaagaagctaaatgtttatactgatagccgttatgcttttgctact
gcccatatccatggagaaatatacagaaggcgtgggtggctcacatcagaaggcaaagagatcaaaaataaaga
cgagatcttggccctactaaaagccctctttctgcccaaaagacttagcataatccattgtccaggacatcaaaggg
acacagcgccgaggctagaggcaaccggatggctgaccaagcggcccgaaaggcagccatcacagagactcc
agacacctctaccctcctcatagaaaattcatcaccctctggcggctcaaaaagaaccgccgacggcagcgaattc
gagcccaagaagaagaggaaagtctaa

PE2-C_Rad51

NLS
Cas9 H840A
Linker A
Linker B
Rad51 ssDBD
Reverse transcriptase

atgaaacggacagccgacggaagcgagttcgagtcaccaaagaagaagcggaaagtcgacaagaagtacagc
atcggcctggacatcggcaccaactctgtgggctgggccgtgatcaccgacgagtacaaggtgcccagcaagaaa
ttcaaggtgctgggcaacaccgaccggcacagcatcaagaagaacctgatcggagccctgctgttcgacagcggc
gaaacagccgaggccacccggctgaagagaaccgccagaagaagatacaccagacggaagaaccggatctg
ctatctgcaagagatcttcagcaacgagatggccaaggtggacgacagcttcttccacagactggaagagtccttcct
ggtggaagaggataagaagcacgagcggcaccccatcttcggcaacatcgtggacgaggtggcctaccacgaga
agtaccccaccatctaccacctgagaaagaaactggtggacagcaccgacaaggccgacctgcggctgatctatct

ggccctggcccacatgatcaagttccggggccacttcctgatcgagggcgacctgaaccccgacaacagcgacgt
ggacaagctgttcatccagctggtgcagacctacaaccagctgttcgaggaaaaccccatcaacgccagcggcgtg
gacgccaaggccatcctgtctgccagactgagcaagagcagacggctggaaaatctgatcgcccagctgcccggc
gagaagaagaatggcctgttcggaaacctgattgccctgagcctgggcctgacccccaacttcaagagcaacttcg
acctggccgaggatgccaaactgcagctgagcaaggacacctacgacgacgacctggacaacctgctggcccag
atcggcgaccagtacgccgacctgtttctggccgccaagaacctgtccgacgccatcctgctgagcgacatcctgag
agtgaacaccgagatcaccaaggcccccctgagcgcctctatgatcaagagatacgacgagcaccaccaggacc
tgaccctgctgaaagctctcgtgcggcagcagctgcctgagaagtacaaagagattttcttcgaccagagcaagaac
ggctacgccggctacattgacggcggagccagccaggaagagttctacaagttcatcaagcccatcctggaaaag
atggacggcaccgaggaactgctcgtgaagctgaacagagaggacctgctgcggaagcagcggaccttcgacaa
cggcagcatcccccaccagatccacctgggagagctgcacgccattctgcggcggcaggaagatttttacccattcc
tgaaggacaaccgggaaaagatcgagaagatcctgaccttccgcatcccctactacgtgggccctctggccaggg
gaaacagcagattcgcctggatgaccagaaagagcgaggaaaccatcacccccctggaacttcgaggaagtggtg
gacaagggcgcttccgcccagagcttcatcgagcggatgaccaacttcgataagaacctgcccaacgagaaggtg
ctgcccaagcacagcctgctgtacgagtacttcaccgtgtataacgagctgaccaaagtgaaatacgtgaccgagg
gaatgagaaagcccgccttcctgagcggcgagcagaaaaaggccatcgtggacctgctgttcaagaccaaccgg
aaagtgaccgtgaagcagctgaaagaggactacttcaagaaaatcgagtgcttcgactccgtggaaatctccggcg
tggaagatcggttcaacgcctccctgggcacataccacgatctgctgaaaattatcaaggacaaggacttcctggac
aatgaggaaaacgaggacattctggaagatatcgtgctgaccctgacactgtttgaggacagagagatgatcgagg
aacggctgaaaacctatgcccacctgttcgacgacaaagtgatgaagcagctgaagcggcggagatacaccggct
ggggcaggctgagccggaagctgatcaacggcatccgggacaagcagtccggcaagacaatcctggatttcctga
agtccgacggcttcgccaacagaaacttcatgcagctgatccacgacgacagcctgacctttaaagaggacatcca
gaaagcccaggtgtccggccagggcgatagcctgcacgagcacattgccaatctggccggcagccccgccattaa
gaagggcatcctgcagacagtgaaggtggtggacgagctcgtgaaagtgatgggccggcacaagcccgagaac
atcgtgatcgaaatggccagagagaaccagaccacccagaagggacagaagaacagccgcgagagaatgaa
gcggatcgaagagggcatcaaagagctgggcagccagatcctgaaagaacaccccgtggaaaacacccagctg
cagaacgagaagctgtacctgtactacctgcagaatgggcgggatatgtacgtggaccaggaactggacatcaac
cggctgtccgactacgatgtggacgctatcgtgcctcagagctttctgaaggacgactccatcgacaacaaggtgctg
accagaagcgacaagaaccggggcaagagcgacaacgtgccctccgaagaggtcgtgaagaagatgaagaa
ctactggcggcagctgctgaacgccaagctgattacccagagaaagttcgacaatctgaccaaggccgagagagg
cggcctgagcgaactggataaggccggcttcatcaagagacagctggtggaaacccggcagatcacaaagcacg
tggcacagatcctggactcccggatgaacactaagtacgacgagaatgacaagctgatccgggaagtgaaagtga
tcaccctgaagtccaagctggtgtccgatttccggaaggatttccagttttacaaagtgcgcgagatcaacaactacca
ccacgcccacgacgcctacctgaacgccgtcgtgggaaccgccctgatcaaaaagtaccctaagctggaaagcg
agttcgtgtacggcgactacaaggtgtacgacgtgcggaagatgatcgccaagagcgagcaggaaatcggcaag
gctaccgccaagtacttcttctacagcaacatcatgaactttttcaagaccgagattaccctggccaacggcgagatcc
ggaagcggcctctgatcgagacaaacggcgaaaccggggagatcgtgtgggataagggccgggattttgccacc
gtgcggaaagtgctgagcatgccccaagtgaatatcgtgaaaaagaccgaggtgcagacaggcggcttcagcaa
agagtctatcctgcccaagaggaacagcgataagctgatcgccagaaagaaggactgggaccctaagaagtacg

gcggcttcgacagccccaccgtggcctattctgtgctggtggtggccaaagtggaaaagggcaagtccaagaaact
gaagagtgtgaaagagctgctggggatcaccatcatggaaagaagcagcttcgagaagaatcccatcgactttctg
gaagccaagggctacaaagaagtgaaaaaggacctgatcatcaagctgcctaagtactccctgttcgagctggaa
aacggccggaagagaatgctggcctctgccggcgaactgcagaagggaaacgaactggccctgccctccaaata
tgtgaacttcctgtacctggccagccactatgagaagctgaagggctcccccgaggataatgagcagaaacagctgt
ttgtggaacagcacaagcactacctggacgagatcatcgagcagatcagcgagttctccaagagagtgatcctggc
cgacgctaatctggacaaagtgctgtccgcctacaacaagcaccgggataagcccatcagagagcaggccgaga
atatcatccacctgtttaccctgaccaatctgggagcccctgccgccttcaagtactttgacaccaccatcgaccggaa
gaggtacaccagcaccaaagaggtgctggacgccaccctgatccaccagagcatcaccggcctgtacgagacac
ggatcgacctgtctcagctgggaggtgactctggaggatctagcggaggatcctctggcagcgagacaccaggaac
aagcgagtcagcaacaccagagagcagtggcggcagcagcggcggcagcagcaccctaaatatagaagatga
gtatcggctacatgagacctcaaaagagccagatgtttctctagggtccacatggctgtctgattttcctcaggcctggg
cggaaaccgggggcatgggactggcagttcgccaagctcctctgatcatacctctgaaagcaacctctacccccgtg
tccataaaacaataccccatgtcacaagaagccagactggggatcaagccccacatacagagactgttggaccag
ggaatactggtaccctgccagtcccccctggaacacgcccctgctacccgttaagaaaccagggactaatgattatag
gcctgtccaggatctgagagaagtcaacaagcgggtggaagacatccaccccaccgtgcccaacccttacaacct
cttgagcgggctcccaccgtcccaccagtggtacactgtgcttgatttaaaggatgcctttttctgcctgagactccaccc
caccagtcagcctctcttcgcctttgagtggagagatccagagatgggaatctcaggacaattgacctggaccagact
cccacagggtttcaaaaacagtcccaccctgtttaatgaggcactgcacagagacctagcagacttccggatccagc
acccagacttgatcctgctacagtacgtggatgacttactgctggccgccacttctgagctagactgccaacaaggtac
tcgggccctgttacaaaccctagggaacctcgggtatcgggcctcggccaagaaagcccaaatttgccagaaaca
ggtcaagtatctggggtatcttctaaaagagggtcagagatggctgactgaggccagaaaagagactgtgatgggg
cagcctactccgaagacccctcgacaactaagggagttcctagggaaggcaggcttctgtcgcctcttcatccctggg
tttgcagaaatggcagcccccctgtaccctctcaccaaaccggggactctgtttaattggggcccagaccaacaaaa
ggcctatcaagaaatcaagcaagctcttctaactgccccagccctggggttgccagatttgactaagccctttgaactct
ttgtcgacgagaagcagggctacgccaaaggtgtcctaacgcaaaaactgggaccttggcgtcggccggtggccta
cctgtccaaaaagctagacccagtagcagctgggtggccccttgcctacggatggtagcagccattgccgtactga
caaaggatgcaggcaagctaaccatgggacagccactagtcattctggcccccatgcagtagaggcactagtca
aacaacccccgaccgctggctttccaacgcccggatgactcactatcaggccttgcttttggacacggaccgggtcc
agttcggaccggtggtagccctgaacccggctacgctgctcccactgcctgaggaagggctgcaacacaactgcctt
gatatcctggccgaagcccacggaacccgacccgacctaacggaccagccgctcccagacgccgaccacacct
ggtacacggatggaagcagtctcttacaagagggacagcgtaaggcgggagctgcggtgaccaccgagaccga
ggtaatctgggctaaagccctgccagccgggacatccgctcagcgggctgaactgatagcactcacccaggcccta
aagatggcagaaggtaagaagctaaatgtttatactgatagccgttatgcttttgctactgcccatatccatggagaaat
atacagaaggcgtgggtggctcacatcagaaggcaaagagatcaaaaataaagacgagatcttggccctactaaa
agccctctttctgcccaaaagacttagcataatccattgtccaggacatcaaaagggacacagcgccgaggctaga
ggcaaccggatggctgaccaagcggcccgaaaggcagccatcacagagactccagacacctctaccctcctcat
agaaaattcatcaccctctggcggctcaaaaagaaccgccgacggcagcgaattcgagTCTGGTGGTTCT
CCCAAGAAGAAGAGGAAAGTCGGTAGTTCCGGATCTgcaatgcagatgcagcttgaagca

aatgcagatacttcagtggaagaagaaagctttggcccacaacccatttcacggttagagcagtgtggcataaatgc
caacgatgtgaagaaattggaagaagctggattccatactgtggaggctgttgcctatgcgccaaagaaggagcta
ataaatattaagggaattagtgaagccaaagctgataaaattctggctgaggcagctaaattagttccaatgggtttca
ccactgcaactgaattccaccaaaggcggtcagagatcatacagattactactggctccaaagagcttgacaaacta
cttcaacccaagaagaagaggaaagtctaa
