# Supplementary material for: Generation of a more efficient prime editor 2 by addition of the Rad51 DNA-binding domain
Source: Nat Commun. 2021 Sep 23;12:5617. doi: 10.1038/s41467-021-25928-2 (PMC8460726; doi:10.1038/s41467-021-25928-2)
Supplement: Supplementary file 2 — Reporting Summary [file 41467_2021_25928_MOESM2_ESM.pdf]

nature research

Corresponding author(s): Hyongbum Henry Kim

Last updated by author(s): Aug 30, 2021

Reporting Summary

Nature Research wishes to improve the reproducibility of the work that we publish. This form provides structure for consistency and transparency in reporting. For further information on Nature Research policies, see our Editorial Policies and the Editorial Policy Checklist.

Statistics

For all statistical analyses, confirm that the following items are present in the figure legend, table legend, main text, or Methods section.

n/a	Confirmed	
☐	☒	The exact sample size (*n*) for each experimental group/condition, given as a discrete number and unit of measurement
☐	☒	A statement on whether measurements were taken from distinct samples or whether the same sample was measured repeatedly
☐	☒	The statistical test(s) used AND whether they are one- or two-sided<br>*Only common tests should be described solely by name; describe more complex techniques in the Methods section.*
☐	☒	A description of all covariates tested
☐	☒	A description of any assumptions or corrections, such as tests of normality and adjustment for multiple comparisons
☐	☒	A full description of the statistical parameters including central tendency (e.g. means) or other basic estimates (e.g. regression coefficient) AND variation (e.g. standard deviation) or associated estimates of uncertainty (e.g. confidence intervals)
☐	☒	For null hypothesis testing, the test statistic (e.g. *F*, *t*, *r*) with confidence intervals, effect sizes, degrees of freedom and *P* value noted<br>*Give P values as exact values whenever suitable.*
☐	☒	For Bayesian analysis, information on the choice of priors and Markov chain Monte Carlo settings
☐	☒	For hierarchical and complex designs, identification of the appropriate level for tests and full reporting of outcomes
☐	☒	Estimates of effect sizes (e.g. Cohen's *d*, Pearson's *r*), indicating how they were calculated

Our web collection on statistics for biologists contains articles on many of the points above.

Software and code

Policy information about availability of computer code

Data collection	No software was used for data collection.
Data analysis	The custom Python (version 3.6.9) script used for prime editing efficiency calculations are available at https://github.com/hkimlab-PE/PE_SupplementaryCode.<br>The Coot program (version WinCoot 0.9.6.1) was used for predicting structures of PE2 and hyPE2.<br>The CueMol (version 2.2.3.443;http://www.cuemol.org) was used to prepare the structural images of PE2 and hyPE2.

For manuscripts utilizing custom algorithms or software that are central to the research but not yet described in published literature, software must be made available to editors and reviewers. We strongly encourage code deposition in a community repository (e.g. GitHub). See the Nature Research guidelines for submitting code & software for further information.

Data

Policy information about availability of data

All manuscripts must include a data availability statement. This statement should provide the following information, where applicable:

- Accession codes, unique identifiers, or web links for publicly available datasets
- A list of figures that have associated raw data
- A description of any restrictions on data availability

The deep sequencing data from this study have been submitted to the National Center for Biotechnology Information Sequence Read Archive under accession number SRP307854.
The protein structure data for predicting structure of PE2 and HyPE2 is from Protein Data Bank (https://www.rcsb.org). PDB code is 6VPC for SpCas9 DNA adenine base editor; 4HKQ for XMRV RT in complex with an RNA:DNA hybrid; 1B22 for N-terminal domain of Rad51; 4OO8 for Cas9; 5DMQ for Reverse transcriptase; 1B22 for Rad51, respectively.

Field-specific reporting

Please select the one below that is the best fit for your research. If you are not sure, read the appropriate sections before making your selection.

[x] Life sciences [ ] Behavioural & social sciences [ ] Ecological, evolutionary & environmental sciences

For a reference copy of the document with all sections, see nature.com/documents/nr-reporting-summary-flat.pdf

Life sciences study design

All studies must disclose on these points even when the disclosure is negative.

Sample size	No statistical methods were used to predetermine sample size. Sample sizes were chosen after deep sequencing, depending on the read number and quality. All sample sizes were sufficient for the model training and for the following statistical tests, as evidenced by the high Spearman correlation coefficient measured for cross-validation and by the statistical values for Student's t-test and one-way ANOVA.
Data exclusions	In order to improve the accuracy of our analysis, pegRNA and target sequence pairs with deep sequencing read counts below 100 were excluded.
Replication	The high-throughput evaluation were independently repeated three times for library A, and two times for library B and linker variants. All replications showed similar results.<br>The individual evaluation experiments of HEK293T cells, HCT116 cells, and human fibroblasts were independently repeated three times, with comparable results.
Randomization	We randomly selected 107 plasmids from a 55K library (previously published library) by colony picking and mixed the selected plasmids at an equimolar ratio (library A).<br>To design library B, we selected 100 deletion-, 100 insertion-, and 200 substitution-inducing pegRNAs from the previously published library of 54,836 pairs of pegRNA-encoding and target sequences. For this selection, we divided the editing efficiencies from the previous study into eight strata (<1%, 1~3%, 3~6%, 6~10%, 10~20%, 20~30%, 30~40%, >40%) and randomly selected a similar number of pegRNAs from each stratum, so that pegRNAs associated with all levels of efficiency would be included.<br>For individual endogenous experiments, we randomly selected 78 plasmids from library A and B by colony picking.
Blinding	Blinding is not relevant to our study, because our study did not require subjective judgment and interpretation. The results presented here are purely based on objective description of our experiments.

Reporting for specific materials, systems and methods

We require information from authors about some types of materials, experimental systems and methods used in many studies. Here, indicate whether each material, system or method listed is relevant to your study. If you are not sure if a list item applies to your research, read the appropriate section before selecting a response.

Materials & experimental systems

n/a	Involved in the study
[x]	[ ] Antibodies
[ ]	[x] Eukaryotic cell lines
[x]	[ ] Palaeontology and archaeology
[x]	[ ] Animals and other organisms
[ ]	[x] Human research participants
[x]	[ ] Clinical data
[x]	[ ] Dual use research of concern

Methods

n/a	Involved in the study
[x]	[ ] ChIP-seq
[x]	[ ] Flow cytometry
[x]	[ ] MRI-based neuroimaging

Eukaryotic cell lines

Policy information about cell lines

Cell line source(s)	HEK293T;American Type Culture Collection (ATCC)<br>HCT116;Korean Cell Line Bank (KCLB)
Authentication	Not been authenticated.
Mycoplasma contamination	Not been tested.
Commonly misidentified lines<br>(See ICLAC register)	No commonly misidentified cell lines were used.

Human research participants

Policy information about studies involving human research participants

Population characteristics	A 32 year-old male adult who does not have any diseases
Recruitment	The healthy individuals were informed about the procedures and the study; those who are willing to participate in this study signed the respective informed written consent.
Ethics oversight	The Institutional Review Board of Severance Hospital, Yonsei University Health System approved the consent procedure and the study (No, 4-2012-0028).

Note that full information on the approval of the study protocol must also be provided in the manuscript.
